# A global real-world study assessing total time to adrenalectomy in primary aldosteronism

**DOI:** 10.1093/ejendo/lvaf124

**Published:** 2025-06-17

**Authors:** Charmaine Ter, Xuan Han Koh, Hieu Tran, Irina Bancos, Mohamed Bassiony, Marta Araujo-Castro, Miguel Paja, Marga González Boillos, Eleftheria Gkaniatsa, Martin Reincke, Christian Adolf, Thang Viet Tran, Michael Stowasser, Drishya Nayak, Marianne A Grytaas, Adina F Turcu, Joanna Matrozova, Norlela Sukor, Farhana Ismail, Tomaz Kocjan, Mirko Parasiliti-Caprino, Rene Baudrand, Thomas Uslar, Mika Tsuiki, Masanori Murakami, Jun Yang, Chrislyn Ng, Takuyuki Katabami, Mitsuhide Naruse, Matthieu St-Jean, Filippo Ceccato, Seyed Ehsan Saffari, Ada E D Teo, Troy H Puar

**Affiliations:** Department of Cardiovascular and Metabolic Disorders, Duke–NUS Medical School, Singapore 169857, Singapore; Department of Endocrinology, Changi General Hospital, Singapore 529889, Singapore; Department of Cardiovascular and Metabolic Disorders, Duke–NUS Medical School, Singapore 169857, Singapore; Department of Endocrinology, Changi General Hospital, Singapore 529889, Singapore; Department of Endocrinology, University of Medicine and Pharmacy at Ho Chi Minh City, Ho Chi Minh City 700000, Vietnam; Division of Endocrinology, Metabolism, Nutrition and Diabetes, Mayo Clinic, Rochester, MN 55905, United States; Department of Medicine, NYC Health + Hospitals/Elmhurst, Icahn School of Medicine at Mount Sinai, New York, NY 10029, United States; Endocrinology and Nutrition Department, Ramón y Cajal University Hospital, 28034 Madrid, Spain; Department of Endocrinology, Basurto University Hospital, 48013 Bilbao, Spain; Endocrinology and Nutrition Department, Hospital Universitario de Castellón, 12004 Castelló de la Plana, Castellón, Spain; Department of Internal Medicine and Clinical Nutrition, Institute of Medicine, Sahlgrenska Academy, University of Gothenburg, 413 90 Gothenburg, Sweden; Department of Medicine IV, LMU Klinikum, Ludwig–Maximilians University, 80539 Munich, Germany; Department of Medicine IV, LMU Klinikum, Ludwig–Maximilians University, 80539 Munich, Germany; Department of Endocrinology, University of Medicine and Pharmacy at Ho Chi Minh City, Ho Chi Minh City 700000, Vietnam; Endocrine Hypertension Research Centre, University of Queensland Frazer Institute, Brisbane, Queensland 4102, Australia; Hypertension Unit, Metro South Health (Princess Alexandra Hospital), Brisbane, Queensland 4102, Australia; Department of Medicine, Haukeland University Hospital, 5021 Bergen, Norway; Division of Metabolism, Endocrinology and Diabetes, Department of Internal Medicine, University of Michigan, Ann Arbor, MI 48109, United States; Department of Endocrinology, University Hospital of Endocrinology–Medical University, 1463 Sofia, Bulgaria; Department of Medicine, Faculty of Medicine, The National University of Malaysia (UKM) Medical Centre, Kuala Lumpur 50300, Malaysia; Department of Medicine, Faculty of Medicine, The National University of Malaysia (UKM) Medical Centre, Kuala Lumpur 50300, Malaysia; Department of Endocrinology, Diabetes and Metabolic Diseases, University Medical Centre Ljubljana, 1000 Ljubljana, Slovenia; Faculty of Medicine, University of Ljubljana, 1000 Ljubljana, Slovenia; Department of Medical Sciences, University of Turin, 10124 Turin, Italy; Department of Endocrinology, Pontificia Universidad Catolica, Santiago 8330074, Chile; Department of Endocrinology, Pontificia Universidad Catolica, Santiago 8330074, Chile; Department of Endocrinology, National Hospital Organization Kyoto Medical Centre, Kyoto 612-0861, Japan; Department of Molecular Endocrinology and Metabolism, Graduate School of Medical and Dental Sciences, Institute of Science Tokyo, 1-5-45 Yushima, Bunkyo-ku, Tokyo 113-8510, Japan; Centre of Endocrinology and Metabolism, Hudson Institute of Medical Research, Clayton, Victoria 3168, Australia; Department of Medicine, Monash University, Clayton, Victoria 3800, Australia; Department of Medicine, Monash University, Clayton, Victoria 3800, Australia; Department of Metabolism and Endocrinology, St. Marianna University Yokohama Seibu Hospital, Kanagawa 241-0811, Japan; Endocrine Centre and Clinical Research Centre, Ijinkai Takeda General Hospital, Kyoto 601-1495, Japan; Division of Endocrinology, Centre de Recherche du Centre Hospitalier Universitaire de Sherbrooke, Sherbrooke, Québec J1H 5N4, Canada; Endocrinology Unit, Department of Medicine DIMED, University of Padova, 35128 Padova, Italy; Centre for Quantitative Medicine, Duke-NUS Medical School, Singapore 169857, Singapore; Department of Neurology, National Neuroscience Institute, Singapore 308433, Singapore; Department of Medicine, Division of Endocrinology, National University Health System, Singapore 119228, Singapore; Department of Cardiovascular and Metabolic Disorders, Duke–NUS Medical School, Singapore 169857, Singapore; Department of Endocrinology, Changi General Hospital, Singapore 529889, Singapore

**Keywords:** global health, primary hyperaldosteronism, endocrine hypertension, diagnostic delay, adrenal vein sampling, functional imaging, adrenal surgery

## Abstract

**Background:**

Primary aldosteronism (PA) is a common treatable cause of hypertension. When caused by unilateral adrenal disease, it is potentially curable by adrenalectomy. However, specialized tests and other factors may delay definitive treatment. We assessed the time to adrenalectomy (TTA) for patients worldwide.

**Methods:**

We conducted an international, multicentre retrospective study involving 39 centres from 15 countries to determine the total time taken from the first presentation to adrenalectomy and the intervals between each stage (screening, confirmatory, subtyping, and adrenalectomy). We included patients with PA who underwent adrenalectomy from January 1, 2018, to October 30, 2022. Post-adrenalectomy outcomes were evaluated using the Primary Aldosteronism Surgery Outcome criteria. We performed multivariable quantile and linear regression to identify characteristics associated with longer TTA.

**Results:**

We included 861 patients, mean age 49.3 ± 11.1 years, and 44.5% were women. Overall median TTA was 13.5 months, IQR: 6.6-24.5. Median intervals were 0.1 months (screening), 1.0 months (confirmatory), 4.1 months (subtyping), and 4.3 months (adrenalectomy). On multivariable analysis, median TTA was increased by 5.4 months for each additional adrenal vein sampling (AVS) procedure. Other factors associated with longer TTA included adrenalectomy post-COVID-19, younger age, and additional screening tests. Compared with countries with routine AVS, those without AVS had a shorter TTA (6.1 vs 15.1 months, *P* < .001), but greater likelihood of absent/partial biochemical success post-adrenalectomy (27.4% vs 12.4%, *P* < .001).

**Conclusion:**

Primary aldosteronism management is time-consuming worldwide, especially for subtyping tests and adrenalectomy. While omitting AVS reduces overall time, patients are less likely to achieve biochemical cure post-adrenalectomy.

## Graphical Abstract

**Graphical Abstract:**
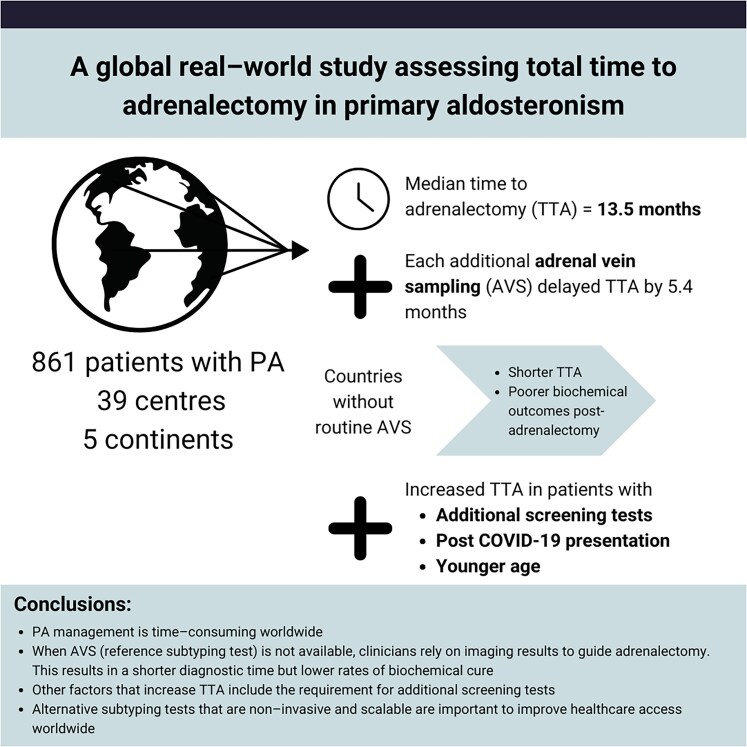

Significance We conducted the first international, multicentre study to determine the total time from the first presentation to adrenalectomy in the management of primary aldosteronism. Most patients required >12 months to be diagnosed and be treated with adrenalectomy. Compared with countries with routine adrenal vein sampling (AVS), countries without AVS had a shorter time to adrenalectomy (TTA), but patients were more likely to have absent/partial biochemical cure post-surgery. Other factors that increased TTA were COVID-19 pandemic, younger age, and additional screening tests. While our study supports the use of AVS, this is not widely available in all countries. Alternative subtyping tests that are non-invasive and scalable are important to improve healthcare access worldwide.

## Introduction

Primary aldosteronism (PA) is a common treatable form of secondary hypertension, affecting 5%-20% of patients with hypertension.^1^ With 1.3 billion adults suffering from hypertension worldwide,^2^ that equates to 64-256 million individuals with PA. Due to the deleterious effects of hyperaldosteronism, patients with PA are at greater risk of cardiovascular and renal complications and suffer from poorer quality of life.^3,4^ One-third of patients have unilateral PA, and laparoscopic unilateral adrenalectomy offers the opportunity to cure hypertension and better long-term outcomes compared with lifelong medications.^4^ Before curative adrenalectomy, current guidelines recommend 3 essential steps (screening, confirmatory, and subtyping) to confirm unilateral PA.^5^ However, each step is fraught with challenges, and healthcare access worldwide may be limited.

Screening and confirmatory tests require aldosterone and renin assays, which may not be available in routine laboratories.^6^ Computed tomography (CT) imaging is unreliable for diagnosing unilateral PA because non-functional adenomas are common, and small aldosterone-producing adenomas (APAs) may be missed. Adrenal vein sampling (AVS) remains the gold standard for subtyping. However, AVS is invasive, technically challenging,^7^ and not widely available, especially in low-resourced countries.^6^ Even where facilities are available, there may be delays in healthcare delivery due to various factors: patient preparation to remove interfering antihypertensive medications, correction of hypokalemia,^5^ and waiting lists for AVS and adrenalectomy.

Currently, there is limited published data on the time taken for each step in the management of PA, differences between countries, and effects of the COVID-19 pandemic. Therefore, our primary objective was to assess the total time required from presentation to adrenalectomy across centres worldwide. Our secondary objectives were to ascertain factors contributing to increased time and to evaluate if the availability of AVS affected biochemical and clinical outcomes of patients.

## Methods

### Study design and patient population

We conducted an international multicentre retrospective study of patients with PA who underwent complete unilateral adrenalectomy. We approached 121 centres worldwide to participate in the study, to achieve a broad geographical representation, with 39 centres responding to participate. Inclusion criteria were all consecutive patients with a confirmed diagnosis of PA in accordance with the Endocrine Society^5^ or Japanese guidelines^8^ who underwent complete adrenalectomy from January 1, 2018, to October 30, 2022. Since AVS was not available in all centres, we included all patients who underwent adrenalectomy, regardless of subtype test performed. The overall study was approved by SingHealth Centralised Institutional Review Board (reference 2023/2054) and conducted in accordance with the Declaration of Helsinki. Each participating centre obtained approval from their local ethics committee and obtained patient consent as required. Patient-level data collected included patient demographics, medication history, biochemical tests, and dates for all endocrine tests and adrenalectomy. The use of antihypertensive medications was expressed as defined daily dose (https://atcddd.fhi.no/atc_ddd_index/).

### Outcomes

The overall time to adrenalectomy (TTA) was taken from the date of the first presentation to the date of adrenalectomy.^5^ The date of the first presentation was defined as the first clinic visit to the PA study centre, first visit to PA specialist, or the date of first screening test, whichever was earliest. The time interval for the screening stage was taken from the date of the first presentation to the date of screening test. The time intervals for each of the other stages (confirmatory, subtyping, and adrenalectomy) were similarly determined (Appendix S1 p. 4).

We evaluated for factors affecting overall TTA, and each interval, and included potential factors previously reported.^9,10^ Additionally, we assessed for the impact of healthcare resourcing on TTA,^11^ by using the World Health Organization index on each country's current health expenditure (CHE) per capita. This was expressed in international purchasing power parity (PPP) dollars.^12^ A higher CHE per capita indicates greater resourcing for healthcare infrastructures.

Patients were evaluated post-adrenalectomy using the Primary Aldosteronism Surgery Outcomes (PASO) criteria for clinical and biochemical outcomes.^10^ We used the clinical and biochemical data obtained at least 30 days post-adrenalectomy in all patients and performed sensitivity analyses after restricting to patients with data at least 180 days post-adrenalectomy.

### Statistical analysis

Continuous variables were compared using Welch's analysis of variance, Mann–Whitney *U*-test or Kruskal–Wallis test (as appropriate). *χ*^2^ test was used for categorical variables (Appendix S1 p. 5). Univariable quantile regression was used to identify patient and centre characteristics associated with TTA at the 25th, 50th, and 75th percentiles (Appendix S1 p. 7-8). Multivariable quantile regression was used to adjust for potential confounders. Potential explanatory characteristics were selected based on existing literature and expert opinion.^9,10^ We performed simultaneous, forced entry of these characteristics into the multivariable model, to minimize bias in the estimated coefficients. Standard errors were obtained via 1000 bootstrap replications. Where there was evidence of non-linearity, we fitted a 2-line piecewise regression using linear splines.

We also performed natural log-transformed TTA to assess if the above results were robust to the analysis method. We presented the 25th, 50th, and 75th percentile TTA as this is more clinically informative than the natural log-transformed mean. For the subgroup analyses, we performed multivariable linear regression of natural log-transformed TTA and TTA intervals to maximize power. The exponentiated, adjusted coefficients and the corresponding exponentiated 95% CIs were reported.

Countries/centres were classified as “non-AVS” if majority (>75%) of patients did not undergo AVS prior to adrenalectomy. Although AVS is recommended for patients being considered for surgery, it may be omitted for some patients such as those younger than 35 years or having a severe PA phenotype, in accordance with the Endocrine Society guidelines. Hence, we defined AVS centres as those where majority (>75%) of patients underwent AVS. In the 4 non-AVS countries, the rates of AVS were <1% (Vietnam, Bulgaria, and Chile) and 48% (Spain).

To compare clinical and biochemical outcomes between AVS countries vs non-AVS countries, we performed multivariable logistic regression with robust standard errors. Statistical tests were 2 sided with a 0.05 significance level. All analyses were conducted using R version 4.4.0 and Stata 18 (College Station, TX, United States: StataCorp LLC).

## Results

We included 861 patients with PA, mean age 49.3 ± 11.1 years, 383 (44.5%) females, who underwent unilateral adrenalectomy from 39 centres in 15 countries. At presentation, of 851 patients with available biochemical data, 437 (51.4%) patients had hypokalaemia. Compared with patients in 11 countries with routine AVS (*N* = 656), those from 4 countries (*N* = 205) without routine AVS were more likely female (62% vs 39%) and had higher mean systolic blood pressure (BP) (151.4 vs 147.9 mmHg, *P* = .031) while on fewer daily-defined dosages of antihypertensive medications (2.3 vs 3.0, *P* = .004). Patients from countries without routine AVS also exhibited a more severe phenotype, with a higher baseline aldosterone–renin ratio (ARR) (132.4 vs 68.5 ng/dL per ng/mL/h, *P* < .001), lower potassium levels (3.3 vs 3.6 mmol/L, *P* < .001), and a lower CHE per capita in PPP ($2553 vs $6722, *P* < .001) (Table 1).

**Table 1.** Characteristics of patients with an adrenalectomy between January 1, 2018, and October 30, 2022, by whether the country routinely performed AVS.

Characteristic	All patients (*n* = 861)	Country routinely performed AVS (*n* = 656)	Country did not routinely perform AVS (*n* = 205)	*P*-value
Demographic				
Age at first presentation^a^ in years, mean ± SD	49.3 ± 11.1	49.7 ± 11.0	48.2 ± 11.3	.108
Female, *n* (%)	383 (44.5)	256 (39.0)	127 (62.0)	<.001
No. of years with hypertension at first presentation^a^, median (IQR)	6 (2-13)	7 (2-13)	6 (2-14)	.347
Clinical, at baseline^b^				
Blood pressure in mmHg, mean ± SD				
Systolic	148.7 ± 19.5	147.9 ± 19.2	151.4 ± 20.4	.031
Diastolic	89.8 ± 12.9	89.4 ± 13.3	91.3 ± 11.7	.051
Hormonal biomarker, median (IQR)				
PAC in ng/dL	28.8 (18.4-44.9)	26.9 (17.7-42)	35.3 (22.1-51.6)	<.001
PRA^c^ in ng/mL/h	0.3 (0.2-0.6)	0.4 (0.2-0.6)	0.2 (0.2-0.3)	<.001
ARR^c^ in ng/dL per ng/mL/h	77.8 (40.8-150.5)	68.5 (34.4-124.1)	132.4 (75.3-202.5)	<.001
Potassium concentration in mmol/L, mean ± SD	3.50 ± 0.64	3.55 ± 0.59	3.31 ± 0.74	<.001
Antihypertensive medication in DDD, median (IQR)	3 (1.5-5.0)	3 (1.7-5)	2.3 (1.3-4)	.004
Adrenal imaging				
Nodule size on first CT scan in mm, median (IQR)	13 (8-19)	12 (5-17)	17 (13-23)	<.001
Nodules on first CT scan, *n* (%)				<.001
None	147/852 (17.3)	142/653 (21.7)	5/199 (2.5)	
Unilateral	618/852 (72.5)	436/653 (66.8)	182/199 (91.5)	
Bilateral	87/852 (10.2)	75/653 (11.5)	12/199 (6.0)	
Centre^d^				
Mean^e^ CHE per capita in PPP (international $), mean ± SD	5730 ± 3157	6722 ± 2883	2553 ± 1422	<.001
Adrenalectomy performed pre-/post-COVID-19^f^, *n* (%)				1.000
Pre	420 (48.8)	320 (48.8)	100 (48.8)	
Post	441 (51.2)	336 (51.2)	105 (51.2)	

To assess differences in characteristics between patients from countries that routinely vs did not routinely perform AVS, the Welch's *t*-test was used for continuous, normally distributed characteristics, the Mann–Whitney *U*-test for continuous, non-normally distributed characteristics, and the *χ*^2^ test for categorical characteristics.

Abbreviations: ARR, aldosterone–renin ratio; AVS, adrenal vein sampling; CHE, current health expenditure; COVID-19, coronavirus disease 2019; DDD, daily defined dose; IQR, interquartile range; PAC, plasma aldosterone concentration; PPP, purchasing power parity; PRA, plasma renin activity; SD, standard deviation.

^a^Date of first the presentation was defined as the earliest of the date of first visit to PA specialist, study centre, or the first screening visit.

^b^Baseline was defined as the measurement pre-adrenalectomy.

^c^Direct renin concentration was converted to plasma renin activity using the following conversion factor: 1 ng/mL/h = 8 ng/dL = 8.2 mU/L. The lower limit of plasma renin activity was fixed at 0.2 ng/mL/h (equivalent to 1.6 ng/dL and 1.64 mU/L).

^d^Where adrenalectomy was performed.

^e^2018-2021.

^f^Before vs after first COVID-19 restriction in district/country that affected the hospital's services.

### Screening, confirmatory, and subtyping tests

Out of 861 patients, 325 (37.7%) had their first screening test performed before their first presentation to the study centre, while 263 (30.5%) had it performed within 2 weeks after presentation. A total of 337 (39.1%) patients had at least 2 screening tests done, while 106 (12.3%) had 3 or more. For confirmatory tests, 233 (27.1%) patients skipped confirmatory tests, 628 (72.9%) patients had at least 1, while 135 (15.7%) had at least 2 confirmatory tests. For subtyping, 683 (79.3%) patients underwent at least 1 AVS, while 36 (4.2%) patients underwent a second AVS. Eighty-seven patients (10.1%) had functional imaging, eg, ^11^C-metomidate positron emission tracer–CT (PET–CT), Gallium-68 Pentixafor PET–CT, I-131-6β-iodomethyl-norcholesterol (NP-59), performed for localization (either in addition or in lieu of AVS).

### Total TTA and each interval between countries

**Figure 1.**
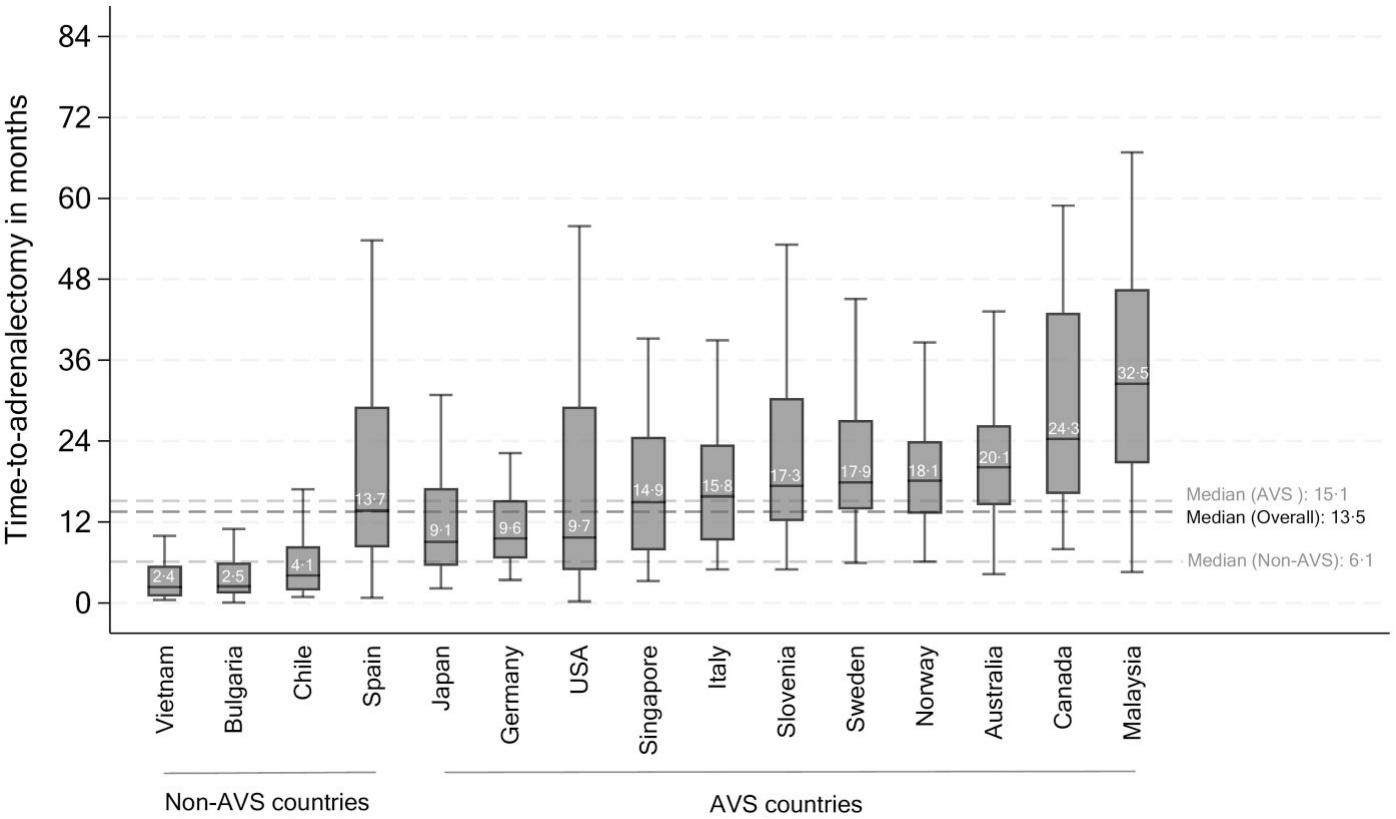
Box plots of TTA, by country of centre where adrenalectomy was performed. Median TTA in months is shown. TTA data more than 1.5 times the interquartile range from the 75th percentile is not shown. “AVS yes” includes countries that routinely perform AVS, while “AVS no” includes countries that do not routinely perform AVS. %”AVS, adrenal vein sampling.

**Figure 2.**
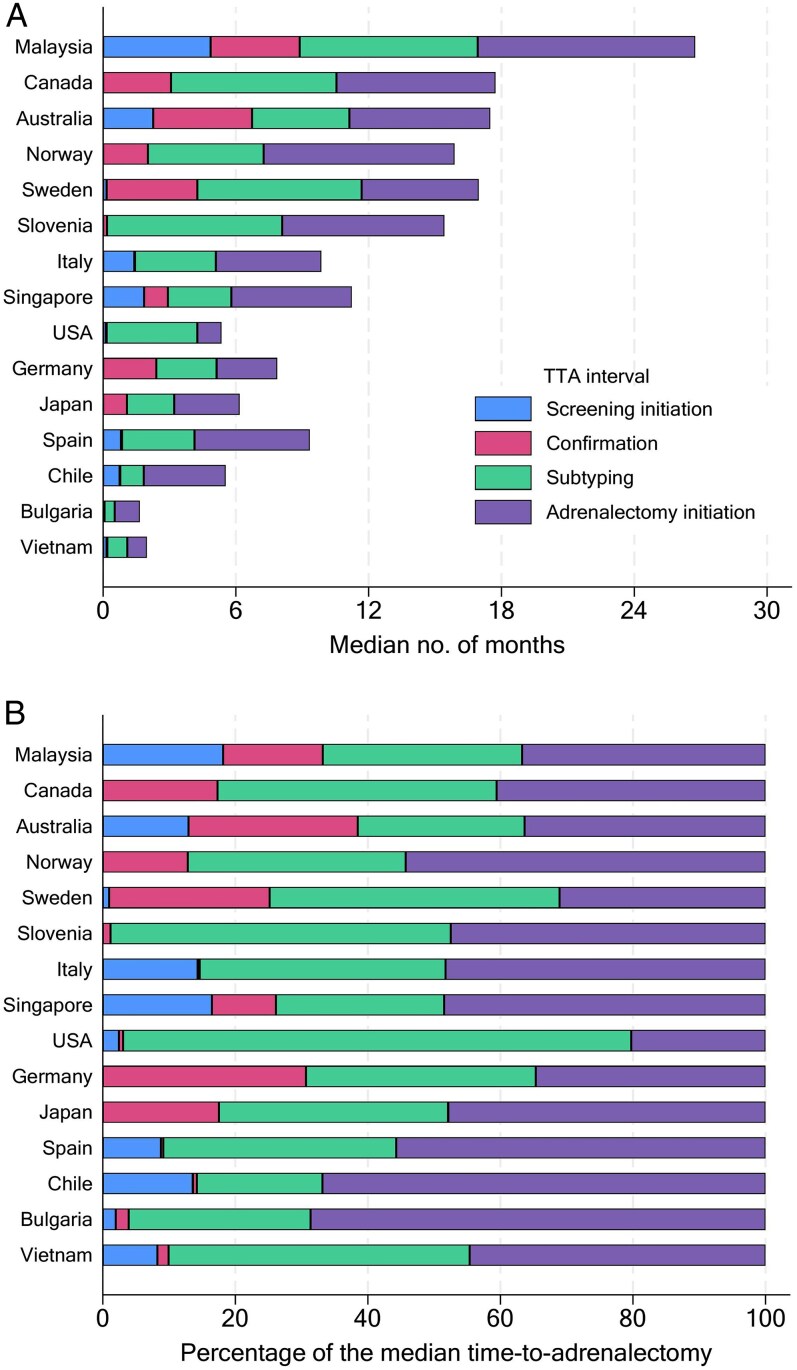
Horizontal stacked bar charts of median TTA intervals, by country of centre where adrenalectomy was performed. (A) Absolute median no. of months. (B) Relative percentage of the median TTA. The sum of the 4 intervals for each country was not equivalent to the median total TTA as different intervals had different participants with missing data and because the sum of medians of intervals does not equal the total median. TTA, time to adrenalectomy.

Among all 861 patients, the median TTA was 13.5 months, IQR: 6.6-24.5 (Figure 1), with a positive skew (Appendix S1 p. 15). Countries without routine AVS had shorter median TTA, 6.1 vs 15.1 months, *P* < .001. Median intervals were 0.1 months for screening, 1.0 months for confirmatory, 4.1 months for subtyping, and 4.3 months for adrenalectomy. Preoperative mineralocorticoid receptor antagonist (MRA) was prescribed in 499 (58.0%) patients, with a median duration of 0.9 months (IQR: 0-4.4). While the contribution to overall TTA by each interval varied between countries (Figure 2A), subtyping and adrenalectomy intervals were the largest contributors to overall TTA in all countries (Figure 2B). Median TTA exceeded 1 year in 9 of 15 countries, while 469 of 861 patients (54.5%) had TTA of more than 1 year and 221 patients (25.7%) had TTA of more than 2 years.

### Factors associated with increased overall TTA

Multivariable quantile regression (Table 2) showed that median TTA was increased by 5.3 months (95% CI, 3.9-6.7, *P* < .001) with each additional screening test, and increased by 5.4 months (95% CI, 2.6-8.3, *P* < .001) with each additional AVS. Median TTA was also increased in patients of younger age, with lower baseline diastolic BP, and those with a smaller nodule size on CT scan. When adrenalectomy was performed post-COVID-19, compared with pre-COVID-19, TTA increased at the 75th percentile by 5.5 months (95% CI, 1.5-9.5, *P* = .007). We found consistent results when we performed multivariable linear regression of natural log-transformed TTA (Appendix S1 p. 9-10). In addition to the above factors, TTA was also increased with more baseline antihypertensive medications. Regarding healthcare expenditure, the 25th percentile and median TTA decreased in countries at both extremes of low and high CHE per capita, while at the 75th percentile, TTA decreased with increasing CHE per capita (Appendix S1 p. 16).

**Table 2.** Multivariable quantile regression of the 25th, 50th, and 75th percentile TTA on patient and country characteristics.

Characteristic	Outcome: 25th percentile TTA in months		Outcome: Median TTA in months		Outcome: 75th percentile TTA in months	
	Adjusted difference in 25th percentiles (95% CI)	*P*-value	Adjusted difference in medians (95% CI)	*P*-value	Adjusted difference in 75th percentiles (95% CI)	*P*-value
Patient						
Ten-year increase in age at first presentation^a^	−0.29 (−0.81 to 0.22)	.267	−1.17 (−2.00 to −0.34)	.006	−2.11 (−3.95 to −0.26)	.025
Female sex	−0.81 (−2.07 to 0.46)	.213	0.08 (−1.76 to 1.92)	.934	−0.16 (−4.47 to 4.15)	.943
Incidentaloma (vs symptomatic) discovery	−0.52 (−1.56 to 0.53)	.330	−0.85 (−2.67 to 0.96)	.356	−3.51 (−7.91 to 0.89)	.118
One mmol/L increase in baseline^b^ potassium	0.64 (−0.25 to 1.54)	.160	0.49 (−1.20 to 2.19)	.568	−0.26 (−3.88 to 3.36)	.889
Ten mmHg increase in baseline^b^ DBP	−0.36 (−0.83 to 0.10)	.128	−0.66 (−1.30 to −0.02)	.042	−1.63 (−3.44 to 0.17)	.076
One DDD increase in baseline^b^ antihypertensive medication	0.09 (−0.16 to 0.34)	.479	0.41 (−0.01 to 0.84)	.057	0.85 (−0.15 to 1.86)	.097
Baseline^b^ ARR^c^ spline term		.459		.502		.316
ARR_1_	−0.13 (−0.33 to 0.07)	.193	−0.23 (−0.65 to 0.19)	.287	−0.66 (−1.53 to 0.20)	.133
ARR_2_	0.21 (−0.13 to 0.55)	.227	0.41 (−0.51 to 1.34)	.378	1.36 (−0.36 to 3.08)	.121
One additional screening test	2.78 (1.54-4.01)	<.001	5.29 (3.85 to 6.73)	<.001	5.82 (1.96-9.68)	.003
One additional confirmatory test	−0.77 (−1.83 to 0.29)	.155	−0.04 (−1.69 to 1.61)	.963	2.43 (−0.97 to 5.83)	.162
Ten mm increase in nodule size on first CT scan^d^	−0.75 (−1.25 to −0.25)	.004	−1.12 (−1.99 to −0.25)	.011	1.01 (−1.10 to 3.13)	.348
One additional AVS	4.37 (2.00-6.73)	<.001	5.40 (2.55 to 8.25)	<.001	13.85 (7.51 to 20.20)	<.001
Centre^e^						
Mean^f^ CHE per capita in PPP (international $) spline term		<.001		<.001		.008
CHE per capita_1_	0.92 (0.22-1.62)	.010	0.90 (−0.15 to 1.95)	.093	−2.26 (−4.49 to −0.03)	.047
CHE per capita_2_	−2.36 (−3.25 to −1.47)	<.001	−2.55 (−3.97 to −1.13)	<.001	1.13 (−1.87 to 4.14)	.458
Adrenalectomy post (vs pre) COVID-19^g^	0.62 (−0.43 to 1.66)	.247	1.31 (−0.42 to 3.03)	.138	5.50 (1.52-9.48)	.007

The multivariable analysis included 795/861 (92.3%) patients with available data for 13 potential explanatory variables that were selected a priori based on the literature and expert clinical opinion. The relationship between TTA with both baseline ARR ratio and mean CHE per capita were modelled using restricted cubic splines with 3 knots at the 10th, 50th, and 90th percentiles.

Abbreviations: ARR, aldosterone–renin ratio; AVS, adrenal vein sampling; CHE, current health expenditure; COVID-19, coronavirus disease 2019; CT, computed tomography; DBP, diastolic blood pressure; DDD, daily defined dose; PPP, purchasing power parity; TTA, time to adrenalectomy.

^a^Date of the first presentation was defined as the earliest of the date of first visit to PA specialist, study centre, or the first screening visit.

^b^Baseline was defined as the measurement pre-adrenalectomy.

^c^Direct renin concentration was converted to plasma renin activity using the following conversion factor: 1 ng/mL/h = 8 ng/dL = 8.2 mU/L. The lower limit of plasma renin activity was fixed at 0.2 ng/mL/h (equivalent to 1.6 ng/dL and 1.64 mU/L).

^d^Larger nodule size was used for bilateral lesions. If there were no lesions, nodule size equalled 0.

^e^Where adrenalectomy was performed.

^f^2018-2021.

^g^After vs before COVID-19 restriction in district/country that affected the hospital's services.

### Comparison between countries with routine AVS vs countries without AVS

**Figure 3.**
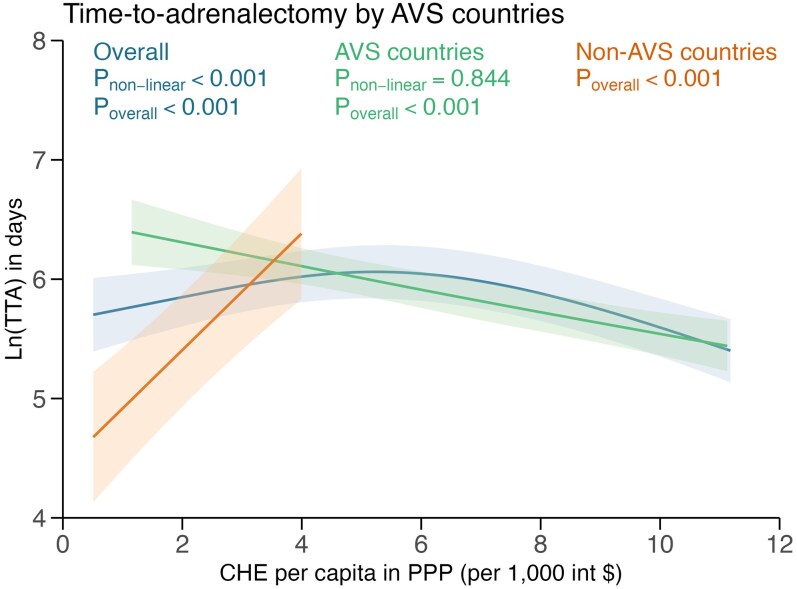
Restricted cubic spline plots of predicted natural log-transformed TTA (line) and 95% CI (area) against the countries’ CHE per capita, by whether country routinely performed AVS. The predicted natural log-transformed TTA values were obtained by fitting a multivariable linear regression. The relationship between natural log-transformed TTA with the countries’ CHE per capita was modelled using restricted cubic splines with 3 knots at the 10th, 50th, and 90th percentiles. We used a linear term only for mean CHE per capita in the “no routine AVS” subgroup as there were only 4 distinct mean CHE per capita values. When plotting splines, all covariates were held constant at their mean or reference level, except the variable of interest. The 95% CIs of predicted natural log-transformed TTA were calculated using the Wald method based on the standard errors of the predictions. AVS, adrenal vein sampling; CHE, current health expenditure; PPP, purchasing power parity; TTA, time to adrenalectomy.

In countries with AVS, longer TTA was associated with younger age, higher baseline ARR, more baseline medications, additional screening tests, and patients with adrenalectomy post-COVID-19 (Table 3). In countries without AVS, an additional screening test and a smaller adrenal nodule on CT scan were associated with longer TTA. While overall TTA was lowest in both extremes of low and high CHE per capita, stratified by AVS availability, there were differences between AVS and non-AVS countries. In non-AVS countries, TTA increased with higher CHE per capita, while in AVS countries, TTA decreased with higher CHE per capita (Figure 3). All participating non-AVS countries had CHE per capita below $5500 and contributed to the overall shorter TTA at low CHE per capita. On multivariable multinomial logistic regression, TTA was not associated with differences in biochemical or clinical outcomes (Appendix S1 p. 13-14).

**Table 3.** Multivariable linear regression of the natural log-transformed TTA, by whether the country routinely performed AVS.

Characteristic	Outcome: Natural log-transformed TTA in days			
	Countries routinely performed AVS (*n* = 617)		Countries did not routinely perform AVS (*n* = 178)	
	Exponentiated, adjusted coefficient (exponentiated 95% CI)	*P*-value	Exponentiated, adjusted coefficient (exponentiated 95% CI)	*P*-value
Patient				
Ten-year increase in age at first presentation^a^	0.87 (0.82-0.93)	<.001	1.07 (0.90-1.27)	.445
Female sex	0.93 (0.81-1.08)	.367	0.85 (0.58-1.24)	.400
Incidentaloma (vs symptomatic) discovery	0.85 (0.71-1.01)	.068	0.85 (0.58-1.23)	.384
One mmol/L increase in baseline^b^ potassium	1.02 (0.88-1.17)	.797	1.04 (0.78-1.39)	.771
Ten mmHg increase in baseline^b^ DBP	0.95 (0.90-1.01)	.091	0.92 (0.79-1.08)	.317
One DDD increase in baseline^b^ antihypertensive medication	1.03 (1.00-1.06)	.049	0.98 (0.90-1.07)	.659
Baseline^b^ ARR^c^ spline term		.016		.837
ARR_1_	0.99 (0.96-1.01)	.419	0.99 (0.95-1.04)	.746
ARR_2_	1.02 (0.99-1.06)	.195	1.01 (0.95-1.07)	.851
One additional screening test	1.36 (1.27-1.46)	<.001	3.14 (1.14-8.64)	.027
One additional confirmatory test	1.01 (0.91-1.13)	.806	1.13 (0.79-1.64)	.497
Ten mm increase in nodule size on first CT scan^d^	0.94 (0.88-1.01)	.105	0.80 (0.66-0.96)	.019
Centre^e^				
Mean^f^ CHE per capita, PPP		<.001		
CHE per capita_1_ (spline term 1)	0.90 (0.85-0.96)	.001	—	—
CHE per capita_2_ (spline term 2)	1.01 (0.90-1.14)	.844	—	—
One thousand international dollars increase (linear)	—	—	1.63 (1.39-1.91)	<.001
Adrenalectomy post (vs pre) COVID-19^g^	1.15 (1.01-1.31)	.040	0.96 (0.66-1.40)	.828

The multivariable subgroup analysis of patients from countries that routinely vs did not routinely perform AVS included 617/656 (94.1%) and 178/205 (86.8%) patients, respectively, with available data for 12 potential explanatory variables that were selected a priori based on the literature and expert clinical opinion. The relationship between TTA with both baseline ARR ratio and mean CHE per capita was modelled using restricted cubic splines with 3 knots at the 10th, 50th, and 90th percentiles. We used a linear term only for mean CHE per capita in the “country did not routinely perform AVS” subgroup as there were only 4 distinct mean CHE per capita values.

Abbreviations: ARR, aldosterone–renin ratio; AVS, adrenal vein sampling; CHE, current health expenditure; COVID-19, coronavirus disease 2019; CT, computed tomography; DBP, diastolic blood pressure; DDD, daily defined dose; PPP, purchasing power parity; TTA, time to adrenalectomy.

^a^Date of the first presentation was defined as the earliest of the date of first visit to PA specialist, study centre, or the first screening visit.

^b^Baseline was defined as the measurement pre-adrenalectomy.

^c^Direct renin concentration was converted to plasma renin activity using the following conversion factor: 1 ng/mL/h = 8 ng/dL = 8.2 mU/L. The lower limit of plasma renin activity was fixed at 0.2 ng/mL/h (equivalent to 1.6 ng/dL and 1.64 mU/L).

^d^Larger nodule size was used for bilateral lesions. If there were no lesions, nodule size equalled 0.

^e^Where adrenalectomy was performed.

^f^2018-2021.

^g^After vs before COVID-19 restriction in district/country that affected the hospital's services.

Patients managed in AVS countries had better biochemical outcomes, with 430 (87.6%) having complete success, 26 (5.3%) partial, 35 (7.1%) absent, compared with outcomes in patients managed in non-AVS countries, with 77 (72.6%) complete, 11 (10.4%) partial, and 18 (17.0%) absent biochemical success, *P* < .001 (Appendix S1 p. 17). On multivariable analysis after adjusting for potential confounders including age at first presentation, sex, baseline potassium, systolic BP, daily defined dose, aldosterone renin ratio, nodule size on first CT scan, mean CHE per capita from 2018 to 2021, and adrenalectomy post- (vs pre) COVID-19, patients managed in a country without AVS were associated with 87% lower odds of complete vs partial/absent biochemical success (OR 0.13, 95% CI, 0.1-0.3, *P* < .001), and 85% lower odds of complete/partial vs absent biochemical success (OR 0.15, 95%, CI 0.1-0.4, *P* < .001), compared with patients in countries with AVS.

In terms of clinical outcomes, patients managed in a country with AVS had similar clinical outcomes, with 146 (25.1%) complete, 298 (51.3%) partial, and 137 (23.6%) absent, compared with patients managed in a country without AVS, 52 (34.2%), 69 (45.4%), and 31 (20.4%) respectively, *P* = .080. The proportion of patients with absent clinical cure and absent/partial biochemical success was 23 of 482 (4.8%) in AVS countries, compared with 8 of 103 (7.8%) in non-AVS countries, *P* = .218. On multivariable analysis, after adjusting for potential confounders, being managed in a country without AVS was not associated with differences in the odds of complete vs partial/absent success (OR 1.16, 95%, CI 0.7-2.1, *P* = .621), or complete/partial vs absent success (OR 0.90, 95% CI, 0.5-1.7, *P* = .747). Our findings for biochemical and clinical PASO outcomes were similar after restricting analyses to only patients with data at least 180 days post-adrenalectomy (Appendix S1 p. 6, 18).

### Characteristics associated with longer TTA intervals

In our exploratory analysis, additional screening tests were associated with longer confirmation, subtyping, and adrenalectomy intervals (Appendix S1 p. 11-12). Having adrenalectomy post-COVID-19 was associated with longer screening and subtyping intervals. In addition, longer screening interval was associated with younger age, lower baseline potassium, and lower baseline diastolic BP. Longer confirmation interval was associated with male gender, symptomatic presentation, and those with fewer baseline antihypertensive medications. Longer subtyping interval was associated with more baseline medications. Longer adrenalectomy initiation interval was associated with lower baseline diastolic BP and smaller nodule size on initial CT scan. Compared with countries in the middle range of CHE per capita, countries with low or high CHE per capita were associated with shorter confirmatory and adrenalectomy intervals, but longer screening intervals.

## Discussion

While unilateral PA is a potentially curable cause of hypertension, we report that most patients worldwide require more than a year to undergo adrenalectomy. Although AVS is a prerequisite subtype test for most patients with PA, AVS was not available in several countries. Even when available, patients faced a significant waiting time before AVS and adrenalectomy, and this was aggravated by the COVID-19 pandemic. While the TTA was markedly shorter in non-AVS countries, patients were less likely to achieve biochemical cure post-surgery. Other factors contributing to increased overall TTA included patients who required repeated screening tests, younger age, lower diastolic BP, and those on more baseline antihypertensive medications. Current guidelines for PA recommend 3 steps prior to adrenalectomy: screening, confirmatory, and subtyping. However, our study demonstrates that these recommended tests and treatment require a significant amount of time to be completed. As many as 20% of patients with hypertension, or 0.3 billion individuals worldwide^1,13^ suffer from PA, but only 1% of all patients are currently diagnosed.^14^ Our study highlights the urgent need for more healthcare resources to diagnose and treat patients with PA.

Each AVS procedure was associated with a longer median TTA by 5 months, while TTA was 9 months longer in AVS countries compared with non-AVS countries. Previous studies have highlighted various challenges faced with AVS, such as the differences in protocols, interpretation, and technical difficulties.^7^ Our study with real-world data highlights another challenge: even when available, arranging an AVS is time-consuming. This was further aggravated during the COVID-19 period, particularly as AVS requires inpatient hospitalization, utilization of angiography suites, and highly skilled personnel.^15^ While AVS is recommended to confirm lateralization, we found that numerous countries lack such services and routinely proceeded to adrenalectomy using CT findings. Compared with AVS countries, patients managed in non-AVS countries less frequently achieved complete biochemical cure, consistent with a previous multicentre, retrospective study comparing AVS-guided vs CT-guided adrenalectomy.^16^ However, our study and previous studies have consistently shown similar clinical benefit in BP and reduction in medications regardless of whether AVS is implemented or not.^16,17^ This could be because in non-AVS countries, the criterion for adrenalectomy is more stringent, and only patients with severe PA phenotype, large nodules on CT scan, and higher likelihood of clinical improvement post-surgery are offered surgery. In our study, all patients who underwent surgery without AVS had a visible nodule (≥10 mm in diameter) on CT imaging, except for 2 patients with a visible nodule on CT that were <10 mm in diameter. This could also be due to decreasing the burden of aldosterone excess in patients with bilateral adrenal pathology.

In most countries, arranging an adrenalectomy contributed the most towards the overall duration (average 4 months). In one previous study, 42.9% of 84 patients^18^ with an adrenal tumour underwent surgery more than 1 year after initial CT imaging, which highlighted both the diagnostic delays and the time required to arrange and wait for surgery. The COVID-19 pandemic contributed to a longer overall TTA, particularly the screening and subtyping intervals. This could be due to repeated closures of hospital facilities worldwide, with disruption to diagnostic workup for many diseases.^19^ While COVID-19 affected various healthcare conditions, its effect is likely more pronounced on non-emergency conditions like PA. Even up to today, hospitals worldwide are still catching up with the backlog of cases caused by COVID-19.^20^ During treatment or investigation delays, it is important that patients receive adequate medical treatment to ameliorate the harmful effects of hyperaldosteronism. In our study, about half of the patients received MRA treatment for a median duration of 1 month. MRA treatment may interfere with biochemical tests and treatment is generally started only after completing subtyping tests but this may not be always necessary or possible.^21^ If adrenalectomy were to be deferred by more than 3 months, it would be prudent to initiate patients on medical treatment with MRA.

Due to significant intra-individual variability in ARR,^22^ patients with PA may have completely normal aldosterone levels on some days.^23^ Hence, repeat testing may be required to diagnose PA, but doing this routinely may add to an increased diagnostic interval. It is recommended that hormonal evaluation should be done after correction of hypokalaemia and removal of interfering medications.^5^ However, this can also lead to diagnostic delay. We found that patients with lower baseline potassium, more baseline antihypertensive medications, and those requiring repeated screening tests had longer TTA. In our study, 37.7% of patients already had screening tests performed prior to their first visit to the study centre. This likely led to an underestimation of the screening interval in our study. If most patients require a medication washout, overall TTA is likely to be even longer.

In patients with a smaller or absent adrenal nodule on CT imaging, TTA was longer in non-AVS countries. This may be due to more uncertainty by patients and clinicians in the indication for adrenalectomy. Other factors that prolonged TTA included a lower diastolic BP and younger age. Since PA is more prevalent in patients with severe or resistant hypertension,^24^ there may be a lower index of suspicion in patients with lower BP. Interestingly, although a younger age is often thought as a risk factor for PA, those of younger age had a longer screening interval. This may be possibly explained by less urgency in managing younger individuals. Some other factors we found in our exploratory analyses, such as male gender resulting in longer confirmation interval, may not have a clear explanation and need to be confirmed in future studies.

We found that overall TTA was shorter in countries with the lowest, and highest, CHE per capita. A higher CHE per capita equates to a higher proportion of resources allocated to healthcare relative to the population size. Equipped with more healthcare resources to manage PA, TTA is expectedly shorter in countries with the highest CHE per capita. This is particularly important for AVS and adrenalectomy as provision of interventional suites, operating theatres, highly skilled proceduralists, and ancillary staff require significant financial support. However, countries with the lowest CHE per capita also had shorter TTA, and these were non-AVS countries. In these countries, it is likely that diagnostic tests are scarce resources that have to be used sparingly. In our study, in non-AVS centres, only 3.4% of patients had a repeated screening test, compared with 50.1% patients from AVS centres. In another study, countries with scarce resources frequently omitted confirmatory testing altogether.^6^ Hence, it is likely that expedient treatment is the priority in these countries, with prompt decisions needed to be made regarding surgery.

With PA estimated to affect a quarter of a billion people worldwide, our study highlights that more needs to be done to address the current gaps in healthcare. In many countries, only a few centres have an excellent AVS service, while in other countries, AVS is not available at all. A recent study has shown that functional imaging with ^11^C-metomidate PET–CT^25,26^ is non-inferior to AVS and offers a non-invasive alternative. However, due to the short half-life of 11-carbon, this limits its exportability. Newer tracers like 68Ga-Pentixafor^27^ are currently being studied and are more scalable. However, these may also require PET–CT facilities that may not be available in centres worldwide. Biomarkers like hybrid hormones may potentially offer a more widely available alternative to identify unilateral subtype, in particular patients with an APA with a *KCNJ5* mutation.^25,28^ Until these subtype tests are widely available, CT imaging may be the only means to identify patients with unilateral PA in countries without AVS. Considering the superior long-term benefits of adrenalectomy compared with medications and the recognition of limited resources, it may be reasonable to offer patients with a florid phenotype and a high probability of clinical cure a choice of adrenalectomy based on CT findings if AVS was not available.

Our study strengths were that this was a large international study with centres from 5 continents represented, offering real-world patient data into the diagnostic and treatment time required for PA. To our knowledge, this is the first study of its kind assessing for total time required for surgical treatment in patients with PA. We also recognized some limitations of our study. Firstly, we did not include patient information on comorbidities that could contribute to increased time for diagnosis or preoperative optimization. Secondly, some patients were screened for PA prior to referral to the study team centres. Hence, we only estimated the TTA based on available data from the study centres. This may have led to an underestimation of TTA, and the true diagnostic time may be even longer. Thirdly, patients’ choice for delayed treatment could also be a contributing factor that is not accounted for. However, in a previous study of patients with PA, most patients highlighted concerns over delay in their management.^29^ Fourthly, we used data from 30 days post-surgery for PASO assessment. On sensitivity analyses after including only patients with data at least 6 months post-surgery, there were no changes in the results. Fifthly, different aldosterone and renin assays were used in various centres, which may affect direct comparability of assay results. Finally, our study included leading centres with specialists interested in PA. We anticipate that treatment rendered in other centres worldwide may be even more delayed.

In conclusion, our multicentre international study demonstrates that the total time required for patients with unilateral PA to eventually undergo adrenalectomy is frequently more than one year. Investigations for PA are often arduous and complex. While AVS is an essential step for subtyping, it is not always available. Having a benchmark now relative to a global median is valuable for physicians and health administrators to manage PA more efficiently. Our study highlights the need for improving healthcare delivery and allocating more resources for management of PA, such as developing infrastructure and training of skilled personnel for AVS and adrenalectomy. Future research should focus on identifying alternative diagnostic approaches and subtyping tests, which may be more scalable and accessible to centres worldwide.

## Supplementary Material

lvaf124_Supplementary_Data
