# Supplementary material for: A global real-world study assessing total time to adrenalectomy in primary aldosteronism
Source: Eur J Endocrinol. 2025 Jun 17;193(1):65–75. doi: 10.1093/ejendo/lvaf124 (PMC12224190; doi:10.1093/ejendo/lvaf124)
Supplement: lvaf124_Supplementary_Data [file lvaf124_supplementary_data.docx]

**SUPPLEMENTARY CONTENTS**

Contents (p. 1)

Time-To-Adrenalectomy (TTA) Investigators (p. 2)

Abbreviations (p. 3)

Definitions of the dates and intervals (p. 4)

Supporting methods (p. 5)

Supporting results (p. 6)

Table S1: Univariable quantile regression of the 25^th^, 50^th^ and 75^th^ percentile time-to-adrenalectomy on patient and centre characteristics (p. 7-8)

Table S2: Univariable and multivariable linear regression of natural log-transformed mean time-to-adrenalectomy on patient and centre characteristics (p. 9-10)

Table S3: Association between patient/centre characteristics and the time-to-adrenalectomy intervals (p. 11-12)

Table S4: Association between time-to-adrenalectomy and primary aldosteronism surgical outcomes (p. 13)

Table S5: Association between time-to-adrenalectomy and primary aldosteronism surgical outcomes for patients with a minimum 180 days between adrenalectomy date and post-adrenalectomy blood pressure date (clinical success) or potassium, aldosterone, and renin date (biochemical success) (p. 14)

Figure S1: Histogram of time-to-adrenalectomy for all 861 patients (15 countries) (p. 15)

Figure S2: Restricted cubic spline plots of predicted time-to-adrenalectomy and 95% confidence interval against the countries’ current health expenditure per capita from 2018 to 2021 (p. 16)

Figure S3: Horizontal stacked bar charts of percentage of patients with complete, partial, or absent success, by country of centre where adrenalectomy was performed (p. 17)

Figure S4: Horizontal stacked bar charts of percentage of patients with complete, partial, or absent success, by country of centre where adrenalectomy was performed, for patients with a minimum 180 days between adrenalectomy date and post-adrenalectomy blood pressure (or potassium, aldosterone, and renin) date (p. 18)

**Time-To-Adrenalectomy (TTA) Investigators**

**Charmaine Ter**, **MD**, Duke-NUS Medical School, Singapore

**Koh Xuan Han, MPH**, Changi General Hospital, Singapore

**Hieu Tran, MD, MSc**, Duke–NUS Medical School, Singapore, Changi General Hospital, Singapore & University Medical Center, Ho Chi Minh, Vietnam

**Irina Bancos**, **MD**, **MS**, Mayo Clinic, Rochester, Minnesota, USA

**Mohamed Bassiony,** **MD**, Department of Medicine, NYC Health + Hospitals / Elmhurst, Icahn School of Medicine at Mount Sinai, New York, USA

**Marta Araujo-Castro, MD, PhD**, Endocrinology & Nutrition Department, Ramón y Cajal University Hospital, Madrid, Spain

**Miguel Paja,** **MD, PhD**, Basurto University Hospital, Bilbao, Spain

**Marga González Boillos,** **MD, PhD**, Hospital General Universitari de Castelló, Castellón, Spain

**Eleftheria Gkaniatsa**, **MD, PhD**, Department of Internal Medicine and Clinical Nutrition, Institute of Medicine, Sahlgrenska Academy, University of Gothenburg, Gothenburg, Sweden

**Martin Reincke, MD**, Department of Medicine IV, LMU Klinikum, Ludwig-Maximilians University, Munich, Germany

**Christian Adolf, MD**, Department of Medicine IV, LMU Klinikum, Ludwig-Maximilians University, Munich, Germany

**Thang Viet Tran, MD, PhD**, University of Medicine and Pharmacy at Ho Chi Minh City, Viet Nam

**Michael Stowasser, MBBS, PhD**, University of Queensland Frazer Institute, Brisbane, Australia

**Drishya Nayak, MBBS**, Metro South Health (Princess Alexandra Hospital), Brisbane, Australia

**Marianne A. Grytaas, MD, PhD**, Department of Medicine, Haukeland University Hospital, Bergen, Norway

**Adina F. Turcu, MD, MS**, Division of Metabolism, Endocrinology and Diabetes, Department of Internal Medicine, University of Michigan, Ann Arbor, Michigan, USA

**Joanna Matrozova, MD, PhD**, University Hospital of Endocrinology-Medical University, Sofia, Bulgaria

**Norlela Sukor, MD, PhD**, Department of Medicine, Faculty of Medicine, The National University of Malaysia (UKM) Medical Center, Kuala Lumpur, Malaysia

**Farhana Ismail, MBBS**, Department of Medicine, Faculty of Medicine, The National University of Malaysia (UKM) Medical Center, Kuala Lumpur, Malaysia

**Tomaz Kocjan, MD, PhD**, Department of Endocrinology, Diabetes and Metabolic Diseases, University Medical Center Ljubljana, Ljubljana, Slovenia & Faculty of Medicine, University of Ljubljana, Ljubljana, Slovenia

**Mirko Parasiliti-Caprino, MD, PhD**, Department of Medical Sciences, University of Turin, Turin, Italy

**Rene Baudrand, MD**, Pontificia Universidad Catolica, Santiago, Chile

**Thomas Uslar, MD**, Pontificia Universidad Catolica, Santiago, Chile

**Mika Tsuiki, MD, PhD**, Department of Endocrinology, National Hospital Organization Kyoto Medical Center, Kyoto, Japan

**Masanori Murakami, MD**, Department of Molecular Endocrinology and Metabolism, Graduate School of Medical and Dental Sciences, Institute of Science Tokyo, Tokyo, Japan

**Jun Yang, MBBS, PhD**, Center of Endocrinology and Metabolism, Hudson Institute of Medical Research, Clayton, Victoria, Australia & Department of Medicine, Monash University, Clayton, Victoria, Australia

**Chrislyn Ng, MD**, Department of Medicine, Monash University, Clayton, Victoria, Australia

**Takuyuki Katabami, MD, PhD**, Department of Metabolism and Endocrinology, St. Marianna University Yokohama Seibu Hospital, Kanagawa, Japan

**Mitsuhide Naruse, MD, PhD**, Endocrine Center and Clinical Research Center, Ijinkai Takeda General Hospital, Kyoto, Japan

**Matthieu St-Jean, MD**, Center de recherche du center hospitalier Universitaire de Sherbrooke, Sherbrooke, Québec, Canada

**Filippo Ceccato, MD, PhD**, University of Padova, Padova, Italy

**Seyed Ehsan Saffari, PhD**, Center for Quantitative Medicine, Duke-NUS Medical School, Singapore & Department of Neurology, National Neuroscience Institute, Singapore

**Ada E.D. Teo, MBBChir(Cantab), PhD,** Department of Medicine, Division of Endocrinology, National University Health System, Singapore

**Troy H Puar, MBBS, PhD**, Changi General Hospital, Singapore & Duke–NUS Medical School, Singapore

We are grateful to the following co-coordinators for their help in this study.

SPAIN-ALDO registry

**Paola Parra Ramírez, MD, PhD**, Endocrinology & Nutrition Department. Hospital Universitario La Paz, Madrid, Spain

**Felicia A. Hanzu, MD, PhD**, Endocrinology & Nutrition Department. Hospital Clinic, Barcelona, Spain

**Abbreviations**

The following abbreviations are used in the tables:

| Abbreviation | Definition |
| --- | --- |
| TTA | Time-to-adrenalectomy |
| AVS | Adrenal vein sampling |
| CT | Computed tomography |
| CCT | Captopril challenge test |
| IV | Intravenous |
| SBP | Systolic blood pressure |
| DBP | Diastolic blood pressure |
| HTN | Hypertension |
| PAC | Plasma aldosterone concentration |
| PRA | Plasma renin activity |
| ARR | Aldosterone-renin ratio |
| DDD | Daily defined dose |
| MRA | Mineralocorticoid receptor antagonist |
| CHE | Current health expenditure |
| PPP | Purchasing power parity |
| COVID-19 | Coronavirus disease 2019 |
| SD | Standard deviation |
| CI | Confidence interval |
| IQR | Interquartile range |
| IRR | Incidence rate ratio |

**Definitions of the dates and intervals**


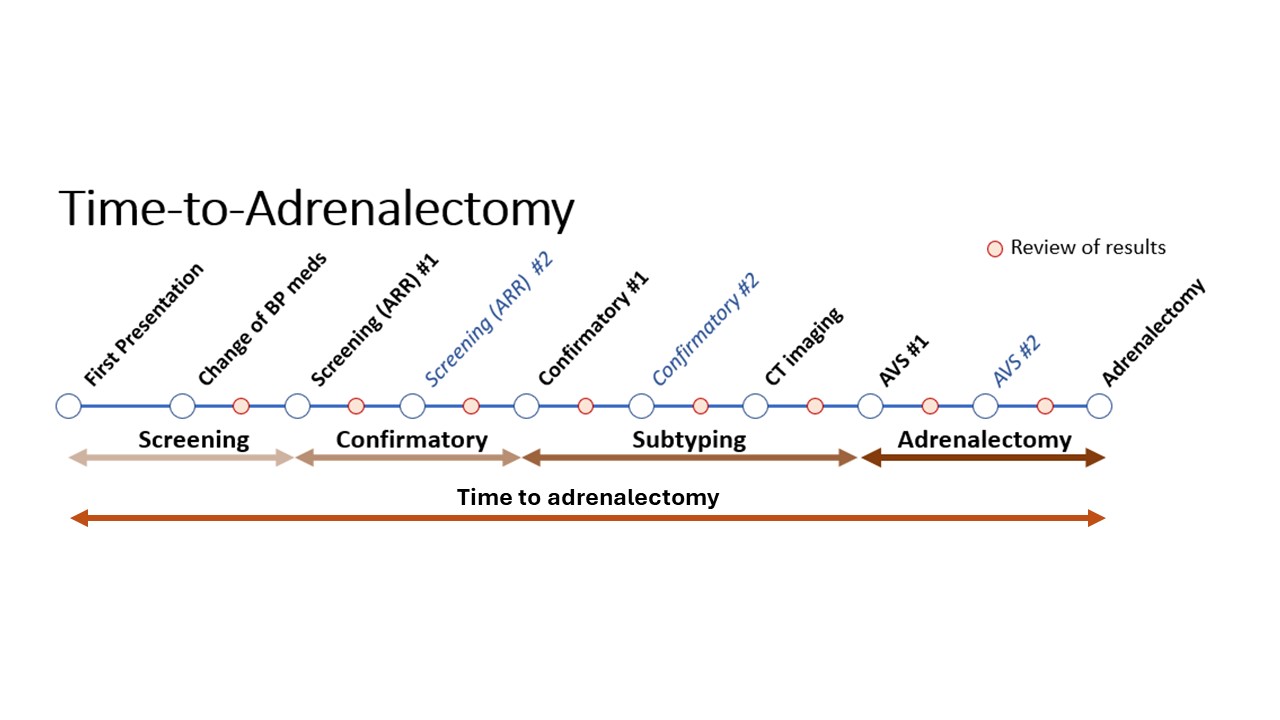


Screening interval was defined as time taken from the first clinic visit to first screening test date with aldosterone–renin ratio (ARR). We excluded participants with a screening test prior to first clinic visit to study centre (e.g. screening test performed by a general practitioner before referring patient to study centre). Confirmation interval was defined as time taken from the first screening test date to first confirmatory test date. Subtyping interval was defined as time taken from the first confirmatory test date to first AVS test date. Adrenalectomy interval was defined as time taken from first AVS test date to adrenalectomy date.

In all patients who omitted AVS, the date of first CT scan was used instead. In all patients who omitted a test, the preceding test was used to calculate the subtyping interval (e.g. in patients who omitted confirmatory test, date of first screening test was used in lieu of confirmatory test). Patients who did not undergo tests in the recommended order (e.g. confirmatory test performed after subtyping test) were excluded from the interval analyses.

Patients who had computed tomography (CT) imaging performed before first presentation to the physician were taken to have presented as ‘incidentaloma’, while the remaining patients were classified as presenting with symptoms. Hypokalaemia was defined as serum potassium of <3·6 mmol/L.

As COVID-19 affected centres at different timings, we gathered responses from all our centres to find out the date of the first restriction in their district or country that affected the centre’s services. This may be defined as any form of disruption to their centre’s typical workflow such as postponing of specialist appointments, limiting manpower, cancellation of elective surgeries. Patients presenting pre COVID-19 were defined as having an adrenalectomy date before the centre’s COVID-19 restriction date. Patients presenting post COVID-19 were defined as having an adrenalectomy date after the centre’s COVID-19 restriction date.

**Supporting methods**

Descriptive statistics of patient demographic and clinical characteristics were reported as number and percent for categorical data, mean ± standard deviation (SD) for normally distributed data, and median and interquartile range (IQR) for non–normally distributed data. To assess between-group differences, the Welch’s t-test or Welch’s ANOVA was used for continuous, normally distributed variables, the Mann-Whitney U-test or Kruskal-Wallis test for continuous, non-normally distributed variables, and the chi-squared test or Fisher’s exact test for categorical characteristics.

We plotted a histogram to visualize the overall TTA distribution. Bin width was selected using the Freeman­–Diaconis rule, i.e. bin width = 2*IQR(TTA)*n^–1/3^. Box plots were used to visualize the TTA distribution by country. Horizontal stacked bar charts illustrated the variation in median TTA intervals across countries, in both absolute and relative (as overall percentage) terms. Horizontal stacked bar charts were used to illustrate PASO outcomes across countries.

Generation of spline plots: Restricted cubic spline plots of model predictions and 95% CIs against CHE per capita were generated. All covariates were held constant at their mean or reference level, except the variable of interest. The 95% CIs of predictions were calculated using the bias-corrected and accelerated (BCa) boostrap method with 1,000 replications (for quantile and zero-inflated negative binomial regression) or the Wald method based on the standard errors of predictions (for linear regression).

Tests of non-linearity and joint contribution of spline terms: Non-linearity and the joint contribution of spline terms were assessed using a likelihood ratio test of the full model with spline terms versus the nested model with the linear term only (or without the variable of interest for overall significance). The Wald test was used to compare quantile regression models.

Two-line piecewise regression with a single change point: Where there was evidence of non-linearity, we fitted a two-line piecewise regression using linear splines. The change point was selected by evaluating CHE per capita values in $500 intervals, with the change point that maximized the model’s log-likelihood being chosen.

A multivariable zero-inflated negative binomial regression with robust standard errors **(Appendix p. 11-12)** was used to model the screening initiation interval as a) 63.9% of patients had a screening initiation interval of zero days, b) there was overdispersion in the positive counts, c) countries with higher mean CHE per capita were thought to have a significantly higher likelihood of an earlier first visit to PA specialist/study centre and thus a non-zero screening initiation interval, and d) it had a non-trivially lower Akaike and Bayesian information criterion compared to a competing hurdle negative binomial-logit regression. For this model, we reported the adjusted incidence rate ratios (IRR) and the 95% CIs.

The Box-Tidwell test assessed if there was evidence of non-linearity between natural log-transformed TTA and continuous covariates for linear regression. A Box-Tidwell test P value <0.05 was taken to suggest violation of the linearity assumption, in which case the affected continuous covariate was categorized (if there were clinically relevant categories), or restricted cubic splines with three knots at the 10th, 50th, and 90th percentiles, were used to model the non-linear relationship. A variance inflation factor of <5 was taken to suggest evidence of multicollinearity. The normality and homoscedasticity assumptions of linear regression were assessed using a quantile–quantile (Q-Q) plot and a plot of residuals against fitted values, respectively. Where there was evidence of heteroscedasticity, robust standard errors were calculated.

**Supporting results**

Analysis of patients with a minimum duration of 180 days from adrenalectomy

Overall, 356 had available data to determine **biochemical success**. Compared to 57 patients in countries without routine AVS, the 299 patients with routine AVS had better biochemical outcomes, 87.3% complete, 5.7% partial, 7.0% absent success vs 70.2% complete, 15.8% partial, 14.0% absent success, P = 0.004. Among 348 patients with available data and a minimum duration of 180 days from adrenalectomy to post-adrenalectomy potassium and ARR measurement, on multivariable analyses after adjusting for potential confounders, patients managed in a country without AVS were associated with a 94% lower odds of complete versus partial/absent success (OR 0.1, 95% CI 0–0.2, P<0.001), and a 92% lower odds of complete/partial versus absent success (OR 0.1, 95% CI 0–0.4, P=0.002), compared to patients in countries with AVS.

Overall, 466 had available data to determine **clinical success**. Compared to 88 patients in countries without routine AVS, the 378 patients with routine AVS had no evidence of a difference in clinical outcomes, 24.9% complete, 51.1% partial, 24.1% absent success vs 33.0% complete, 45.5% partial, 21.6% absent success, P = 0.301. Among 454 patients with available data and a minimum duration of 180 days from adrenalectomy to post-adrenalectomy blood pressure measurement, on multivariable analyses after adjusting for potential confounders, being managed in a country without AVS was not associated with differences in the odds of complete versus partial/absent success (OR 1.3, 95% CI 0.5–3.1, P=0.567), or complete/partial versus absent success (OR 0.9, 95% CI 0.4–2.2, P=0.861).

**Table S1**. Univariable quantile regression of the 25^th^, 50^th^ and 75^th^ percentile time-to-adrenalectomy on patient and centre characteristics.

| **Characteristic** | **Outcome: 25 percentile TTA in months** | | **Outcome: 50^th^ percentile TTA in months** | | **Outcome: 75^th^ percentile TTA in months** | |
| --- | --- | --- | --- | --- | --- | --- |
|  | **Unadjusted difference in 25^th^ percentiles (95% CI)** | **P value** | **Unadjusted difference in 50^th^ percentiles (95% CI)** | **P value** | **Unadjusted difference in 75^th^ percentiles (95% CI)** | **P value** |
| **Patient** |  |  |  |  |  |  |
| Ten-year increase in age at first  presentation* | 0.33 (-0.39 to 1.04) | 0.367 | -0.05 (-1.04 to 0.94) | 0.922 | -2.68 (-4.66 to 0.71) | 0.008 |
| Female sex | -1.15 (-2.74 to 0.44) | 0.156 | -3.02 (-4.80 to -1.25) | 0.001 | -4.27 (-9.08 to 0.53) | 0.081 |
| Incidentaloma (vs. symptomatic) discovery | -2.83 (-4.31 to -1.34) | <0.001 | -4.83 (-7.70 to -1.96) | 0.001 | -8.64 (-13.10 to -4.18) | <0.001 |
| One mmol/L increase in baseline† potassium | 1.37 (0.13 to 2.60) | 0.031 | 2.19 (0.54 to 3.84) | 0.010 | 0.94 (-3.57 to 5.45) | 0.683 |
| Ten mmHg increase in baseline† blood pressure |  |  |  |  |  |  |
| Systolic | -0.15 (-0.54 to 0.23) | 0.431 | -0.31 (-0.98 to 0.37) | 0.374 | -0.36 (-1.84 to 1.11) | 0.629 |
| Diastolic | -0.16 (-0.89 to 0.57) | 0.659 | -1.40 (-1.92 to -0.89) | <0.001 | -0.63 (-2.50 to 1.24) | 0.507 |
| One DDD increase in baseline† antihypertensive medication | 0.17 (-0.01 to 0.36) | 0.065 | 0.23 (-0.37 to 0.82) | 0.454 | 1.24 (0.14 to 2.34) | 0.027 |
| One additional year with HTN at first presentation* | 0.02 (-0.04 to 0.09) | 0.493 | -0.04 (-0.15 to 0.07) | 0.461 | -0.24 (-0.47 to -0.01) | 0.042 |
| Baseline† ARR‡ spline term |  | 0.004 |  | 0.079 |  | 0.227 |
| ARR_1_ | -0.23 (-0.42 to -0.04) | 0.011 | -0.45 (-0.89 to -0.01) | 0.045 | -0.96 (-2.06 to 0.14) | 0.087 |
| ARR_2_ | 0.25 (-0.10 to 0.60) | 0.157 | 0.68 (-0.22 to 1.58) | 0.153 | 1.81 (-0.42 to 4.05) | 0.112 |
| One additional screening test | 4.16 (3.15 to 5.17) | <0.001 | 5.15 (4.22 to 6.07) | <0.001 | 6.98 (2.67 to 11.29) | 0.002 |
| One additional confirmatory test | 1.97 (1.32 to 2.63) | <0.001 | 1.02 (-1.01 to 3.05) | 0.325 | 2.63 (-1.94 to 7.20) | 0.259 |
| Type of 1^st^ confirmatory test |  |  |  |  |  |  |
| Not performed | 0 (ref.) | – | 0 (ref.) | – | 0 (ref.) | – |
| IV saline loading | 7.00 (5.44 to 8.55) | <0.001 | 7.56 (4.59 to 10.52) | <0.001 | 4.37 (-1.25 to 9.99) | 0.127 |
| CCT | 0.07 (-1.11 to 1.24) | 0.912 | -2.10 (-4.97 to 0.76) | 0.150 | -7.62 (-13.03 to -2.21) | 0.006 |
| Others | 2.17 (0.84 to 3.50) | 0.001 | 2.50 (-0.78 to 5.78) | 0.135 | 8.44 (-1.19 to 18.07) | 0.086 |
| Ten mm increase in nodule size on 1^st^ CT scan§ | -1.88 (-2.49 to -1.28) | <0.001 | -2.04 (-2.91 to -1.18) | <0.001 | -2.97 (-4.85 to -1.10) | 0.002 |
| Nodules on 1^st^ CT scan |  |  |  |  |  |  |
| None | 0 (ref.) | – | 0 (ref.) | – | 0 (ref.) | – |
| Unilateral | -4.99 (-7.51 to -2.47) | <0.001 | -5.98 (-9.17 to -2.78) | <0.001 | -5.85 (-13.26 to 1.56) | 0.122 |
| Bilateral | -4.60 (-7.82 to -1.38) | 0.005 | -4.34 (-8.20 to -0.47) | 0.028 | -2.23 (-13.15 to 8.68) | 0.688 |
| One additional AVS | 6.31 (5.22 to 7.40) | <0.001 | 10.15 (8.36 to 11.94) | <0.001 | 13.60 (9.68 to 17.52) | <0.001 |
| Treatment with MRA (vs. no MRA)‖ |  |  |  |  |  |  |
| Preoperative | 1.15 (-0.23 to 2.53) | 0.102 | 0.92 (-1.39 to 3.23) | 0.434 | 2.43 (-2.55 to 7.41) | 0.338 |
| At baseline†, excluding preoperative | -0.72 (-2.56 to 1.12) | 0.441 | 0.56 (-4.10 to 5.22) | 0.814 | 11.56 (1.72 to 21.41) | 0.021 |
| **Centre**¶ |  |  |  |  |  |  |
| Mean** CHE per capita in PPP (international $) spline term |  | <0.001 |  | <0.001 |  | 0.631 |
| CHE per capita_1_ | 2.21 (1.88 to 2.54) | <0.001 | 3.43 (2.63 to 3.86) | <0.001 | 0.61 (-1.56 to 2.77) | 0.583 |
| CHE per capita_2_ | -3.35 (-3.85 to -2.85) | <0.001 | -4.69 (-5.87 to -3.50) | <0.001 | -0.11 (-3.47 to 3.25) | 0.948 |
| Adrenalectomy post (vs. pre) COVID-19†† | 0.99 (-0.39 to 2.37) | 0.161 | 2.40 (0.40 to 4.40) | 0.019 | 6.73 (1.27 to 12.20) | 0.016 |
| One additional week to schedule a clinic |  |  |  |  |  |  |
| Endocrine | 0.30 (0.25 to 0.36) | <0.001 | 0.28 (0.19 to 0.37) | <0.001 | 0.27 (0.10 to 0.44) | 0.002 |
| Surgery | 0.20 (0.13 to 0.27) | <0.001 | 0.15 (-0.03 to 0.33) | 0.095 | 0.09 (-0.24 to 0.43) | 0.585 |

*Date of first presentation was defined as the earliest of the date of first visit to primary aldosteronism specialist, study centre, or the first screening visit.

†Baseline was defined as the measurement pre-adrenalectomy.

‡Direct renin concentration was converted to plasma renin activity using the following conversion factor: 1 ng/mL/hr = 8 ng/dL = 8.2 mU/L. The lower limit of plasma renin activity was fixed at 0.2 ng/mL/hr (equivalent to 1.6 ng/dL and 1.64 mU/L).

§Larger nodule size was used for bilateral lesions. If there were no lesions, nodule size equalled zero.

‖Spironolactone, eplerenone, amiloride, triamterene, esaxerenone, or potassium canrenoate.

¶Where adrenalectomy was performed.

**2018 to 2021.

††After vs. before COVID-19 restriction in district/country that affected the hospital’s services.

ARR=Aldosterone-renin ratio. AVS=Adrenal vein sampling. CCT=Captopril challenge test. CHE=Current health expenditure. CI=Confidence interval. COVID-19=Coronavirus disease 2019. CT=Computed tomography. DDD=Daily defined dose. HTN=Hypertension. IV=Intravenous. MRA=Mineralocorticoid receptor antagonist. PPP=Purchasing power parity. TTA=Time-to-adrenalectomy.

**Table S2**. Univariable and multivariable linear regression of natural log-transformed mean time-to-adrenalectomy on patient and centre characteristics.

| **Characteristic** | **Outcome: Natural log-transformed TTA in days** | | | |
| --- | --- | --- | --- | --- |
|  | **Exponentiated, unadjusted coefficient (exponentiated 95% CI)** | **P value** | **Exponentiated, adjusted coefficient (exponentiated 95% CI)** | **P value** |
| **Patient** |  |  |  |  |
| Ten-year increase in age at first presentation* | 0.99 (0.93 to 1.06) | 0.789 | 0.92 (0.86 to 0.97) | 0.005 |
| Female sex | 0.79 (0.68 to 0.92) | 0.002 | 0.97 (0.84 to 1.12) | 0.647 |
| Incidentaloma (vs. symptomatic) discovery | 0.64 (0.53 to 0.76) | <0.001 | 0.86 (0.72 to 1.01) | 0.070 |
| One mmol/L increase in baseline† potassium | 1.20 (1.05 to 1.38) | 0.007 | 1.05 (0.92 to 1.19) | 0.478 |
| Ten mmHg increase in baseline† blood pressure |  |  |  |  |
| Systolic | 0.98 (0.94 to 1.02) | 0.251 | (Excluded) |  |
| Diastolic | 0.94 (0.89 to 0.99) | 0.033 | 0.94 (0.89 to 0.99) | 0.031 |
| One DDD increase in baseline† antihypertensive medication | 1.04 (1.01 to 1.07) | 0.005 | 1.05 (1.02 to 1.08) | <0.001 |
| One additional year with HTN at first presentation* | 0.99 (0.98 to 1.00) | 0.078 | (Excluded) |  |
| Elevated (vs. non-elevated) baseline† ARR‡ | 1.21 (0.95 to 1.54) | 0.116 | 0.94 (0.76 to 1.17) | 0.594 |
| One additional screening test | 1.44 (1.34 to 1.54) | <0.001 | 1.33 (1.24 to 1.43) | <0.001 |
| One additional confirmatory test | 1.26 (1.12 to 1.41) | <0.001 | 1.04 (0.93 to 1.16) | 0.468 |
| Type of 1^st^ confirmatory test |  | <0.001 | (Excluded) |  |
| Not performed | 1 (ref.) | – |  |  |
| IV saline loading | 1.90 (1.57 to 2.30) | <0.001 |  |  |
| CCT | 0.78 (0.59 to 1.03) | 0.075 |  |  |
| Others | 1.48 (1.17 to 1.87) | 0.001 |  |  |
| Ten mm increase in nodule size on 1^st^ CT scan§ | 0.79 (0.73 to 0.85) | <0.001 | 0.93 (0.86 to 1.00) | 0.066 |
| Nodules on 1^st^ CT scan |  | <0.001 | (Excluded) |  |
| None | 1 (ref.) | – |  |  |
| Unilateral | 0.64 (0.54 to 0.76) | <0.001 |  |  |
| Bilateral | 0.76 (0.58 to 1.00) | 0.048 |  |  |
| One additional AVS | 2.47 (2.07 to 2.95) | <0.001 | 1.82 (1.49 to 2.21) | <0.001 |
| Treatment with MRA (vs. no MRA)‖ |  |  |  |  |
| Preoperative | 1.14 (0.98 to 1.32) | 0.096 | (Excluded) |  |
| At baseline†, excluding preoperative | 1.26 (1.02 to 1.56) | 0.035 | (Excluded) |  |
| **Centre**¶ |  |  |  |  |
| Mean** CHE per capita in PPP (international $) spline term |  | <0.001 |  | <0.001 |
| CHE per capita_1_ | 1.36 (1.26 to 1.46) | <0.001 | 1.11 (1.02 to 1.21) | 0.011 |
| CHE per capita_2_ | 0.66 (0.60 to 0.73) | <0.001 | 0.78 (0.70 to 0.87) | <0.001 |
| Adrenalectomy post (vs. pre) COVID-19†† | 1.20 (1.03 to 1.39) | 0.016 | 1.18 (1.03 to 1.35) | 0.018 |
| One additional week to schedule a clinic |  |  |  |  |
| Endocrine | 1.02 (1.02 to 1.03) | <0.001 | (Excluded) |  |
| Surgery | 1.02 (1.01 to 1.03) | <0.001 | (Excluded) |  |

The multivariable analysis included 795/861 (92.3%) patients with available data for 13 potential explanatory variables that were selected a priori based on the literature and expert clinical opinion. The relationship between natural log-transformed time-to-adrenalectomy and mean CHE per capita was modelled using restricted cubic splines with three knots at the 10th, 50th, and 90th percentiles.

*Date of first presentation was defined as the earliest of the date of first visit to primary aldosteronism specialist, study centre, or the first screening visit.

†Baseline was defined as the measurement pre-adrenalectomy.

‡Based on centre-specific cut-off. The lower limit of plasma renin activity was fixed at 0.2 ng/mL/hr (equivalent to 1.6 ng/dL and 1.64 mU/L).

§Larger nodule size was used for bilateral lesions. If there were no lesions, nodule size equalled zero.

‖Spironolactone, eplerenone, amiloride, triamterene, esaxerenone, or potassium canrenoate.

¶Where adrenalectomy was performed.

**2018 to 2021.

††After vs. before COVID-19 restriction in district/country that affected the hospital’s services.

ARR=Aldosterone-renin ratio. AVS=Adrenal vein sampling. CCT=Captopril challenge test. CHE=Current health expenditure. CI=Confidence interval. COVID-19=Coronavirus disease 2019. CT=Computed tomography. DDD=Daily defined dose. HTN=Hypertension. IV=Intravenous. MRA=Mineralocorticoid receptor antagonist. PPP=Purchasing power parity. TTA=Time-to-adrenalectomy.

**Table S3**. Association between patient/centre characteristics and the time-to-adrenalectomy intervals.

| **Model and characteristic*** | **Screening initiation interval in days (n=431)** | | **Natural log-transformed confirmation interval in days (n=788)** | | **Natural log-transformed subtyping interval in days (n=651)** | | **Natural log-transformed adrenalectomy initiation interval in days (n=727)** | |
| --- | --- | --- | --- | --- | --- | --- | --- | --- |
|  | **Adjusted IRR (95% CI)** | **P value** | **Exponentiated, adjusted coefficient (exponentiated 95% CI)** | **P value** | **Exponentiated, adjusted coefficient (exponentiated 95% CI)** | **P value** | **Exponentiated, adjusted coefficient (exponentiated 95% CI)** | **P value** |
| **Patient** |  |  |  |  |  |  |  |  |
| Ten-year increase in age at first presentation† | 0.70 (0.54 to 0.90) | 0.006 | 0.90 (0.78 to 1.04) | 0.163 | 0.92 (0.84 to 1.02) | 0.107 | 1.02 (0.94 to 1.10) | 0.687 |
| Female sex | 0.73 (0.44 to 1.20) | 0.215 | 0.68 (0.49 to 0.94) | 0.019 | 1.06 (0.86 to 1.30) | 0.591 | 0.88 (0.72 to 1.07) | 0.211 |
| Incidentaloma (vs. symptomatic) discovery | 1.32 (0.74 to 2.36) | 0.349 | 0.38 (0.27 to 0.54) | <0.001 | 1.09 (0.86 to 1.38) | 0.495 | 1.04 (0.83 to 1.30) | 0.734 |
| One mmol/L increase in baseline‡ potassium | 0.59 (0.38 to 0.93) | 0.024 | 0.86 (0.65 to 1.14) | 0.283 | 1.11 (0.92 to 1.33) | 0.284 | 1.11 (0.93 to 1.33) | 0.237 |
| Ten mmHg increase in baseline‡ DBP | 0.68 (0.57 to 0.81) | <0.001 | 1.00 (0.88 to 1.14) | 0.948 | 0.99 (0.91 to 1.07) | 0.788 | 0.88 (0.82 to 0.94) | <0.001 |
| One DDD increase in baseline‡ antihypertensive medication | 1.06 (0.95 to 1.18) | 0.337 | 0.93 (0.87 to 0.99) | 0.028 | 1.07 (1.02 to 1.11) | 0.003 | 1.00 (0.96 to 1.04) | 0.956 |
| Baseline‡ ARR§ spline term | (Excluded) |  | (Excluded) |  |  | 0.532 |  | 0.007 |
| ARR_1_ |  |  |  |  | 1.01 (0.98 to 1.04) | 0.539 | 1.00 (0.97 to 1.03) | 0.827 |
| ARR_2_ |  |  |  |  | 0.98 (0.94 to 1.03) | 0.436 | 1.02 (0.97 to 1.06) | 0.445 |
| One additional screening test | (Excluded) |  | 1.31 (1.06 to 1.60) | 0.011 | 1.37 (1.22 to 1.54) | <0.001 | 1.17 (1.06 to 1.30) | 0.003 |
| One additional confirmatory test | (Excluded) |  | (Excluded) |  | (Excluded) |  | 1.11 (0.98 to 1.27) | 0.111 |
| Ten mm increase in nodule size on 1^st^ CT scan‖ | (Excluded) |  | (Excluded) |  | 0.97 (0.86 to 1.09) | 0.573 | 0.88 (0.79 to 0.97) | 0.010 |
| One additional AVS | (Excluded) |  | (Excluded) |  | (Excluded) |  | (Excluded) |  |
| **Centre**¶ |  |  |  |  |  |  |  |  |
| Mean** CHE per capita in PPP (international $) spline term |  | 0.010 |  | <0.001 |  | 0.012 |  | <0.001 |
| CHE per capita_1_ | 0.75 (0.63 to 0.91) | 0.003 | 1.84 (1.60 to 2.13) | <0.001 | 1.01 (0.88 to 1.15) | 0.937 | 1.28 (1.16 to 1.40) | <0.001 |
| CHE per capita_2_ | 1.66 (1.20 to 2.29) | 0.002 | 0.41 (0.33 to 0.50) | <0.001 | 0.92 (0.76 to 1.10) | 0.335 | 0.50 (0.44 to 0.57) | <0.001 |
| Adrenalectomy post (vs. pre) COVID-19†† | 1.90 (1.14 to 3.16) | 0.014 | 0.77 (0.57 to 1.05) | 0.096 | 1.43 (1.16 to 1.75) | 0.001 | 1.16 (0.97 to 1.38) | 0.100 |

431, 788, 651, and 727 patients had available screening initiation, confirmation, subtyping, and adrenalectomy initiation interval data, respectively, as well as available explanatory variable data. A multivariable zero-inflated negative binomial regression with robust standard errors was used to model the screening initiation interval. Adjusted incidence rate ratios were reported for the count part. All other TTA intervals were modelled using multivariable linear regression after natural log transformation. The exponentiated, adjusted coefficients and corresponding 95% CIs were reported. The relationship between time-to-adrenalectomy with both baseline aldosterone-to-renin ratio and mean current health expenditure per capita were modelled using restricted cubic splines with three knots at the 10th, 50th, and 90th percentiles.

*Characteristics that were not temporally before or during a particular interval were excluded from the multivariable analysis of said interval.

†Date of first presentation was defined as the earliest of the date of first visit to primary aldosteronism specialist, study centre, or the first screening visit.

‡Baseline was defined as the measurement pre-adrenalectomy.

§Direct renin concentration was converted to plasma renin activity using the following conversion factor: 1 ng/mL/hr = 8 ng/dL = 8.2 mU/L. The lower limit of plasma renin activity was fixed at 0.2 ng/mL/hr (equivalent to 1.6 ng/dL and 1.64 mU/L).

‖Larger nodule size was used for bilateral lesions. If there were no lesions, nodule size equalled zero.

¶Where adrenalectomy was performed.

**2018 to 2021.

††After vs. before COVID-19 restriction in district/country that affected the hospital’s services.

ARR=Aldosterone-renin ratio. AVS=Adrenal vein sampling. CHE=Current health expenditure. CI=Confidence interval. COVID-19=Coronavirus disease 2019. CT=Computed tomography. DBP=Diastolic blood pressure. DDD=Daily defined dose. IRR=Incidence rate ratio. PPP=Purchasing power parity. TTA=Time-to-adrenalectomy.

**Table S4**. Association between time-to-adrenalectomy and primary aldosteronism surgical outcomes.

| **Subgroup, success category, and explanatory variable** | **Clinical success** | | | | **Biochemical success** | | | |
| --- | --- | --- | --- | --- | --- | --- | --- | --- |
|  | **Unadjusted relative risk ratio (95% CI)** | **P value** | **Adjusted*** **relative risk ratio (95% CI)** | **P value** | **Unadjusted relative risk ratio (95% CI)** | **P value** | **Adjusted*** **relative risk ratio (95% CI)** | **P value** |
| **All patients** |  |  |  |  |  |  |  |  |
| Complete success | (base outcome) | – | (base outcome) | – | (base outcome) | – | (base outcome) | – |
| Partial success |  |  |  |  |  |  |  |  |
| One-month increase in TTA | 1.01 (1.00 to 1.01) | 0.023 | 1.01 (1.00 to 1.01) | 0.116 | 0.97 (0.94 to 1.01) | 0.206 | 0.98 (0.94 to 1.02) | 0.258 |
| Absent success |  |  |  |  |  |  |  |  |
| One-month increase in TTA | 1.01 (1.00 to 1.01) | 0.149 | 1.01 (1.00 to 1.02) | 0.286 | 1.00 (0.99 to 1.01) | 0.773 | 1.00 (0.99 to 1.01) | 0.566 |
| **Country routinely performed AVS** |  |  |  |  |  |  |  |  |
| Complete success | (base outcome) | – | (base outcome) | – | (base outcome) | – | (base outcome) | – |
| Partial success |  |  |  |  |  |  |  |  |
| One-month increase in TTA | 1.01 (1.00 to 1.01) | 0.124 | 1.01 (1.00 to 1.02) | 0.143 | 0.98 (0.94 to 1.02) | 0.367 | 0.99 (0.95 to 1.02) | 0.382 |
| Absent success |  |  |  |  |  |  |  |  |
| One-month increase in TTA | 1.00 (1.00 to 1.01) | 0.406 | 1.01 (1.00 to 1.02) | 0.188 | 1.00 (0.99 to 1.01) | 0.722 | 1.00 (0.99 to 1.01) | 0.668 |
| **Country did not routinely perform AVS** | |  |  |  |  |  |  |  |
| Complete success | (base outcome) | – | (base outcome) | – | (base outcome) | – | (base outcome) | – |
| Partial success |  |  |  |  |  |  |  |  |
| One-month increase in TTA | 1.01 (1.00 to 1.03) | 0.134 | 1.00 (0.99 to 1.02) | 0.990 | 0.98 (0.95 to 1.01) | 0.116 | 0.99 (0.94 to 1.03) | 0.535 |
| Absent success |  |  |  |  |  |  |  |  |
| One-month increase in TTA | 1.01 (0.99 to 1.03) | 0.310 | 1.00 (0.97 to 1.02) | 0.758 | 1.01 (0.99 to 1.03) | 0.308 | 1.01 (0.98 to 1.03) | 0.562 |

733/861 (85.1%) patients with available blood pressure, anti-hypertensive medication, and at least 30 days between adrenalectomy date and post-adrenalectomy blood pressure date were included in the analysis for clinical success. Of these 733, 581 were from countries that routinely performed AVS, while 152 were from countries that did not routinely perform AVS. 713/733 (97.3%) patients had available data for the ten explanatory variables and were included in the multivariable multinomial logistic regression analysis.

597/861 (69.3%) patients with available potassium, aldosterone, renin, and at least 30 days between adrenalectomy date and post-adrenalectomy potassium, aldosterone, and renin dates were included in the analysis for biochemical success. Of these 597, 491 were from countries that routinely performed AVS, while 106 were from countries that did not routinely perform AVS. 579/597 (97.0%) patients had available data for the ten explanatory variables and were included in the multivariable multinomial logistic regression analysis.

*All multivariable analyses adjusted for continuous age at first presentation, sex, continuous baseline potassium, systolic blood pressure, antihypertensive medication daily defined dose, aldosterone-renin ratio, nodule size on 1^st^ CT scan, mean current health expenditure per capita from 2018 to 2021, and adrenalectomy post (vs. pre) COVID-19.

AVS=Adrenal vein sampling. CI=Confidence interval. TTA=Time-to-adrenalectomy.

**Table S5**. Association between time-to-adrenalectomy and primary aldosteronism surgical outcomes for patients with a minimum 180 days between adrenalectomy date and post-adrenalectomy blood pressure date (clinical success) or potassium, aldosterone, and renin date (biochemical success).

| **Subgroup, success category, and explanatory variable** | **Clinical success** | | | | **Biochemical success** | | | |
| --- | --- | --- | --- | --- | --- | --- | --- | --- |
|  | **Unadjusted relative risk ratio (95% CI)** | **P value** | **Adjusted*** **relative risk ratio (95% CI)** | **P value** | **Unadjusted relative risk ratio (95% CI)** | **P value** | **Adjusted*** **relative risk ratio (95% CI)** | **P value** |
| **All patients** |  |  |  |  |  |  |  |  |
| Complete success | (base outcome) | – | (base outcome) | – | (base outcome) | – | (base outcome) | – |
| Partial success |  |  |  |  |  |  |  |  |
| One-month increase in TTA | 1.01 (1.00 to 1.01) | 0.059 | 1.01 (1.00 to 1.02) | 0.049 | 0.97 (0.92 to 1.03) | 0.329 | 0.97 (0.92 to 1.03) | 0.364 |
| Absent success |  |  |  |  |  |  |  |  |
| One-month increase in TTA | 1.01 (1.00 to 1.02) | 0.079 | 1.01 (1.00 to 1.02) | 0.113 | 1.01 (1.00 to 1.01) | 0.191 | 1.01 (1.00 to 1.02) | 0.066 |
| **Country routinely performed AVS** |  |  |  |  |  |  |  |  |
| Complete success | (base outcome) | – | (base outcome) | – | (base outcome) | – | (base outcome) | – |
| Partial success |  |  |  |  |  |  |  |  |
| One-month increase in TTA | 1.01 (1.00 to 1.01) | 0.107 | 1.01 (1.00 to 1.02) | 0.052 | 0.98 (0.93 to 1.04) | 0.508 | 0.99 (0.95 to 1.03) | 0.569 |
| Absent success |  |  |  |  |  |  |  |  |
| One-month increase in TTA | 1.01 (1.00 to 1.02) | 0.145 | 1.01 (1.00 to 1.03) | 0.063 | 1.01 (1.00 to 1.01) | 0.190 | 1.01 (1.00 to 1.02) | 0.166 |
| **Country did not routinely perform AVS** | |  |  |  |  |  |  |  |
| Complete success | (base outcome) | – | (base outcome) | – | (base outcome) | – | (base outcome) | – |
| Partial success |  |  |  |  |  |  |  |  |
| One-month increase in TTA | 1.01 (0.99 to 1.02) | 0.388 | 1.00 (0.98 to 1.02) | 0.982 | 0.97 (0.94 to 1.00) | 0.082 | 0.92 (0.83 to 1.02) | 0.097 |
| Absent success |  |  |  |  |  |  |  |  |
| One-month increase in TTA | 1.01 (0.99 to 1.02) | 0.372 | 1.00 (0.97 to 1.03) | 0.976 | 1.01 (0.99 to 1.03) | 0.389 | 1.01 (0.97 to 1.05) | 0.652 |

497/861 (57.7%) patients with available blood pressure, anti-hypertensive medication, and at least 180 days between adrenalectomy date and post-adrenalectomy blood pressure date were included in the analysis for clinical success. Of these 497, 409 were from countries that routinely performed AVS, while 88 were from countries that did not routinely perform AVS. 485/497 (97.6%) patients had available data for the ten explanatory variables and were included in the multivariable multinomial logistic regression analysis.

383/861 (44.5%) patients with available potassium, aldosterone, renin, and at least 180 days between adrenalectomy date and post-adrenalectomy potassium, aldosterone, and renin dates were included in the analysis for biochemical success. Of these 383, 326 were from countries that routinely performed AVS, while 57 were from countries that did not routinely perform AVS. 375/567 (96.8%) patients had available data for the ten explanatory variables and were included in the multivariable multinomial logistic regression analysis.

*All multivariable analyses adjusted for continuous age at first presentation, sex, continuous baseline potassium, systolic blood pressure, antihypertensive medication daily defined dose, aldosterone-renin ratio, nodule size on 1^st^ CT scan, mean current health expenditure per capita from 2018 to 2021, and adrenalectomy post (vs. pre) COVID-19.

AVS=Adrenal vein sampling. CI=Confidence interval. TTA=Time-to-adrenalectomy.


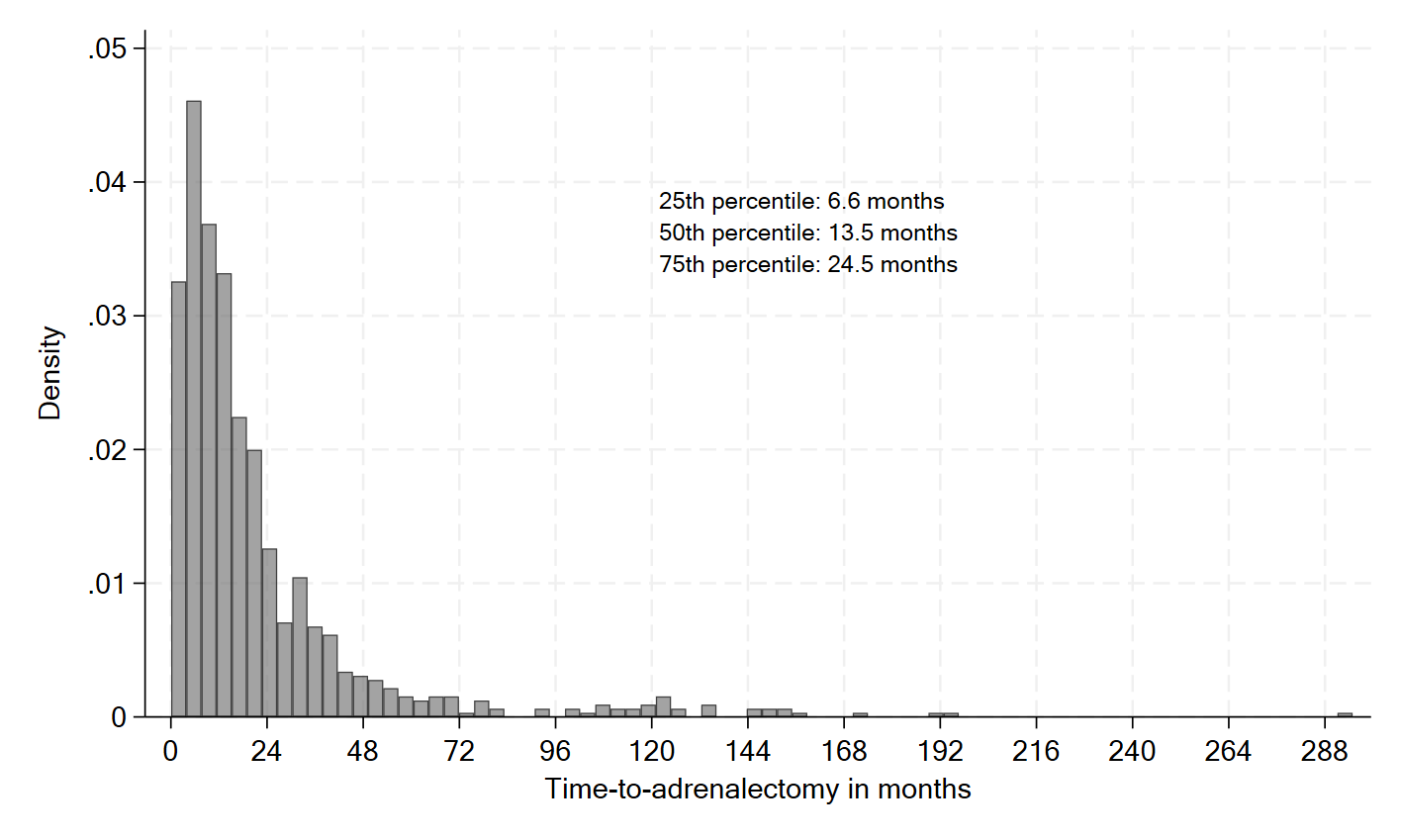


**Figure S1**. Histogram of time-to-adrenalectomy for all 861 patients (15 countries).

Bin width was selected using the Freeman­–Diaconis rule, i.e. bin width = 2*IQR(TTA)*n^-1/3^.


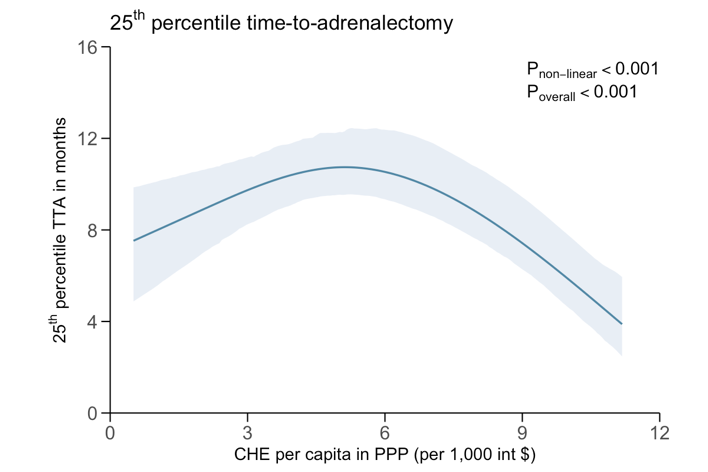

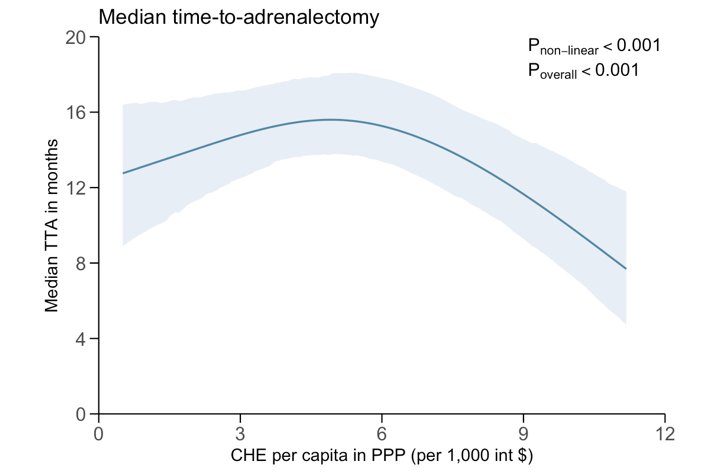

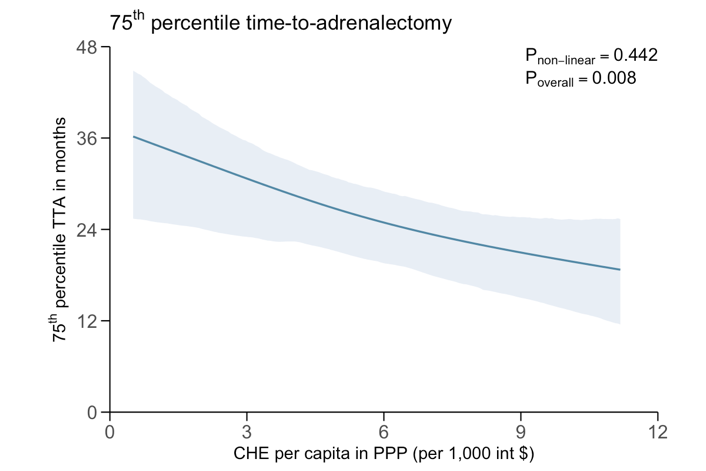


**Figure S2.** Restricted cubic spline plots of predicted time-to-adrenalectomy (blue line) and 95% confidence interval (blue area) against the countries’ current health expenditure per capita from 2018 to 2021.

The predicted time-to-adrenalectomy values were obtained by fitting a multivariable quantile regression, adjusting for age at first presentation, sex, incidentaloma discovery, baseline potassium, baseline diastolic blood pressure, baseline antihypertensive medication, baseline aldosterone-to-renin ratio, no. of screening tests, no. of confirmatory tests, nodule size on 1st computed tomography scan, no. of adrenal vein sampling procedures, country’s current health expenditure per capita, and adrenalectomy post (vs. pre) COVID-19. The relationship between time-to-adrenalectomy with the countries’ current health expenditure per capita was modelled using restricted cubic splines with three knots at the 10th, 50th, and 90th percentiles. When plotting splines, all covariates were held constant at their mean or reference level, except the variable of interest. The 95% confidence intervals of predicted time-to-adrenalectomy were calculated using the bias-corrected and accelerated (BCa) bootstrap method with 1,000 replications.

CHE=Current health expenditure. PPP=Purchasing power parity. TTA=Time-to-adrenalectomy.


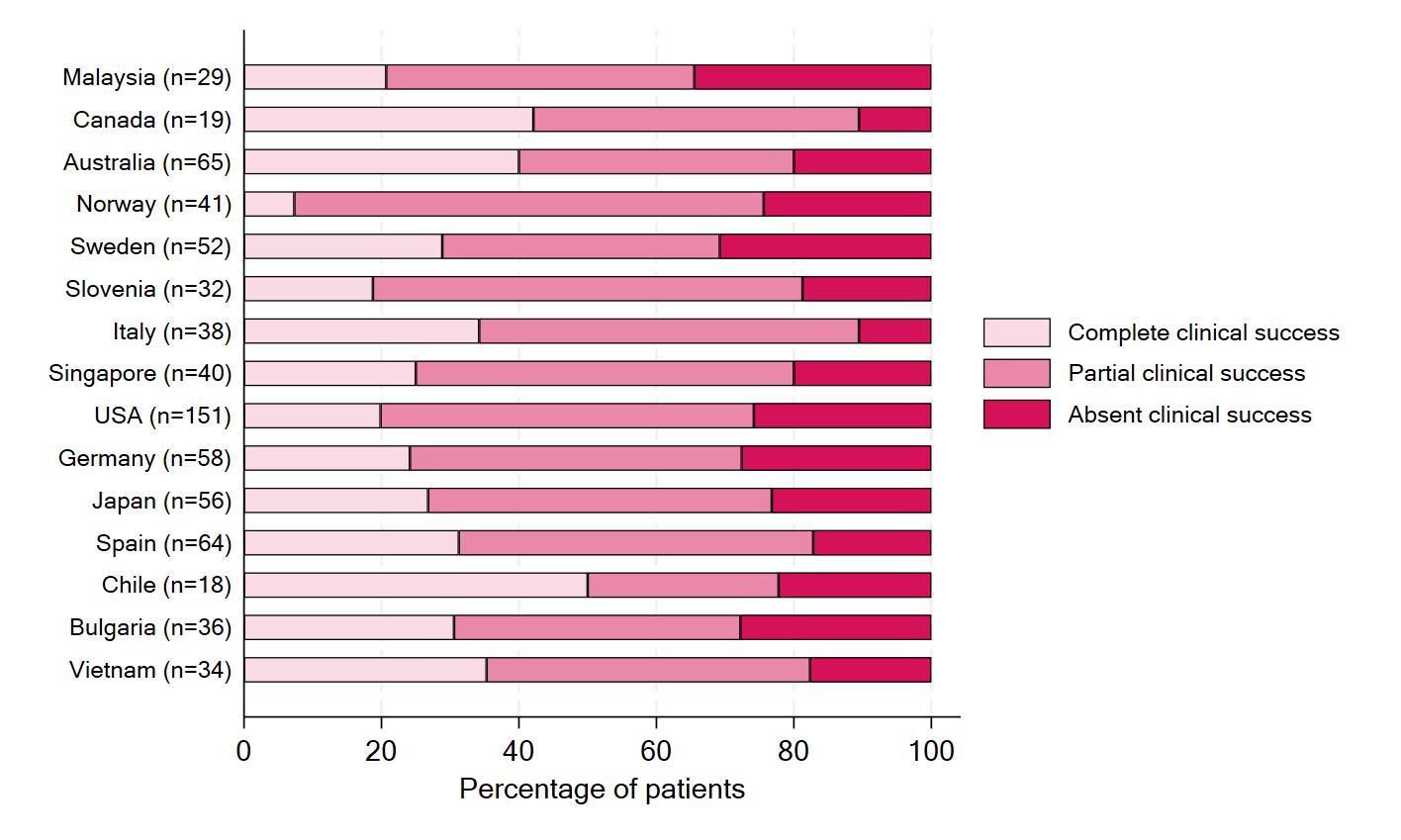


**B**

**A**


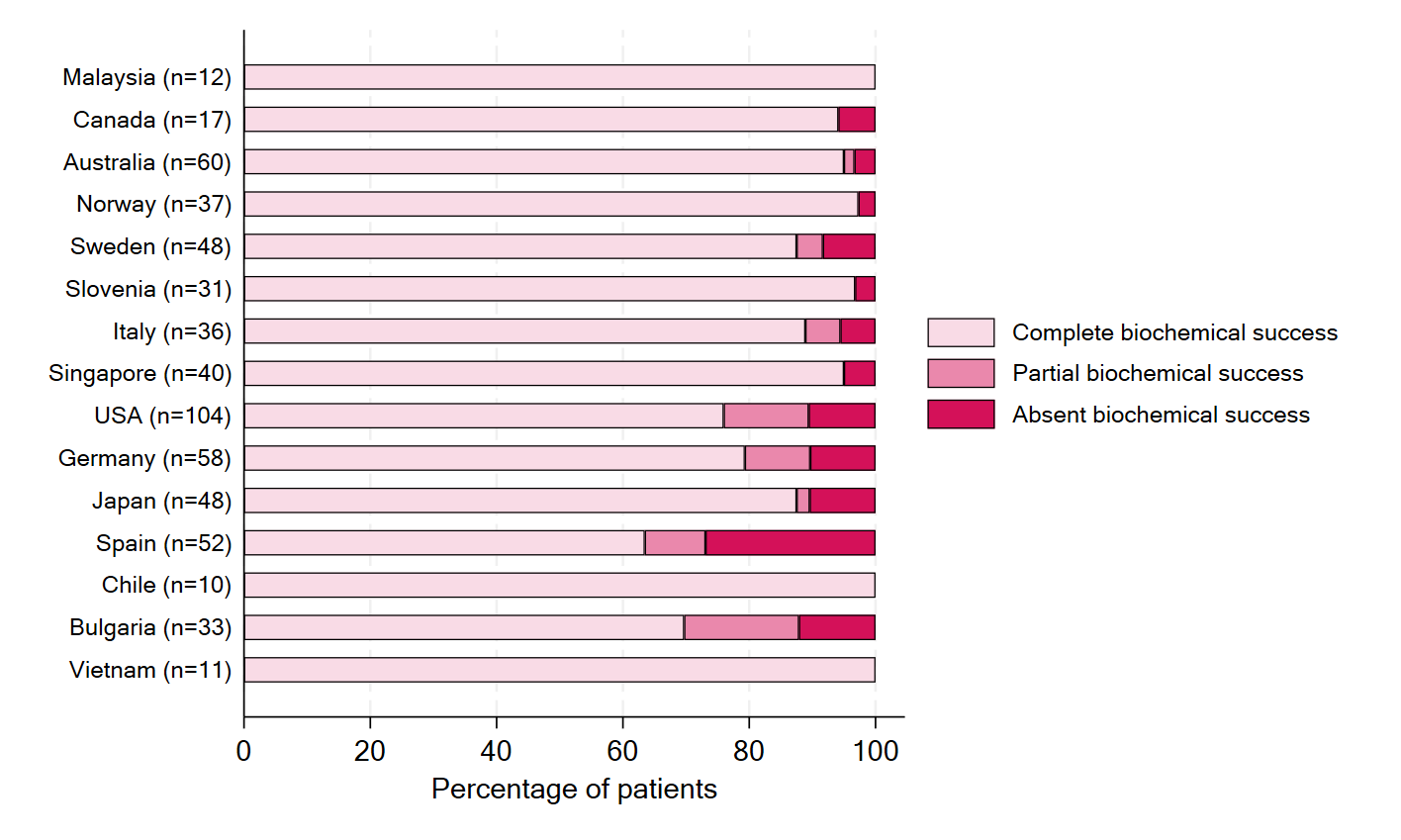


**Figure S3**. Horizontal stacked bar charts of percentage of patients with complete, partial, or absent success, by country of centre where adrenalectomy was performed. **A**. Clinical success. **B**. Biochemical success.

733/861 (85.1%) patients had available blood pressure, anti-hypertensive medication, and at least 30 days between adrenalectomy date and post-adrenalectomy blood pressure date to determine clinical success status. 597/861 (69.3%) patients had available potassium, aldosterone, renin, and at least 30 days between adrenalectomy date and post-adrenalectomy potassium, aldosterone, and renin dates to determine biochemical success status.

USA=United States of America.


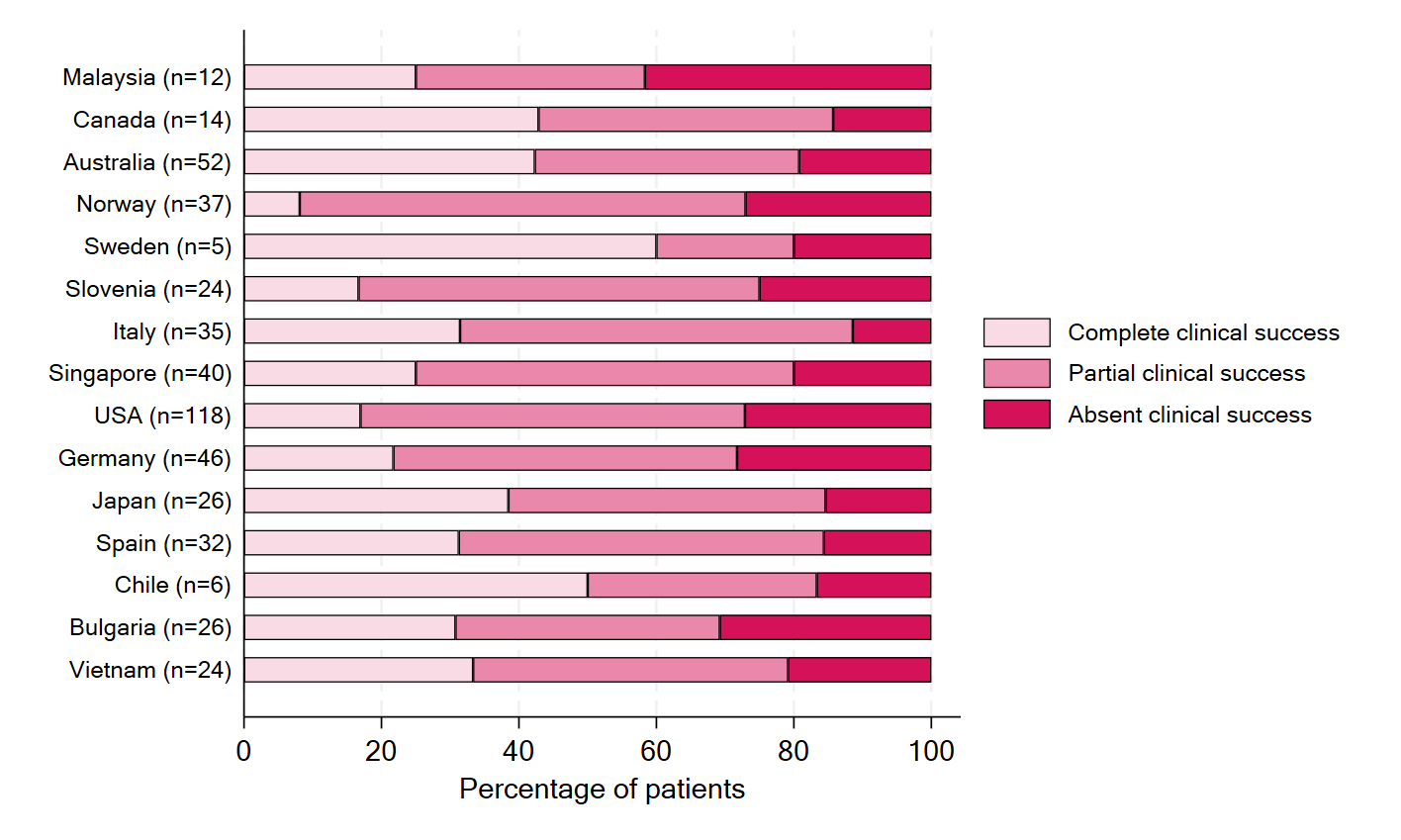


**B**

**A**


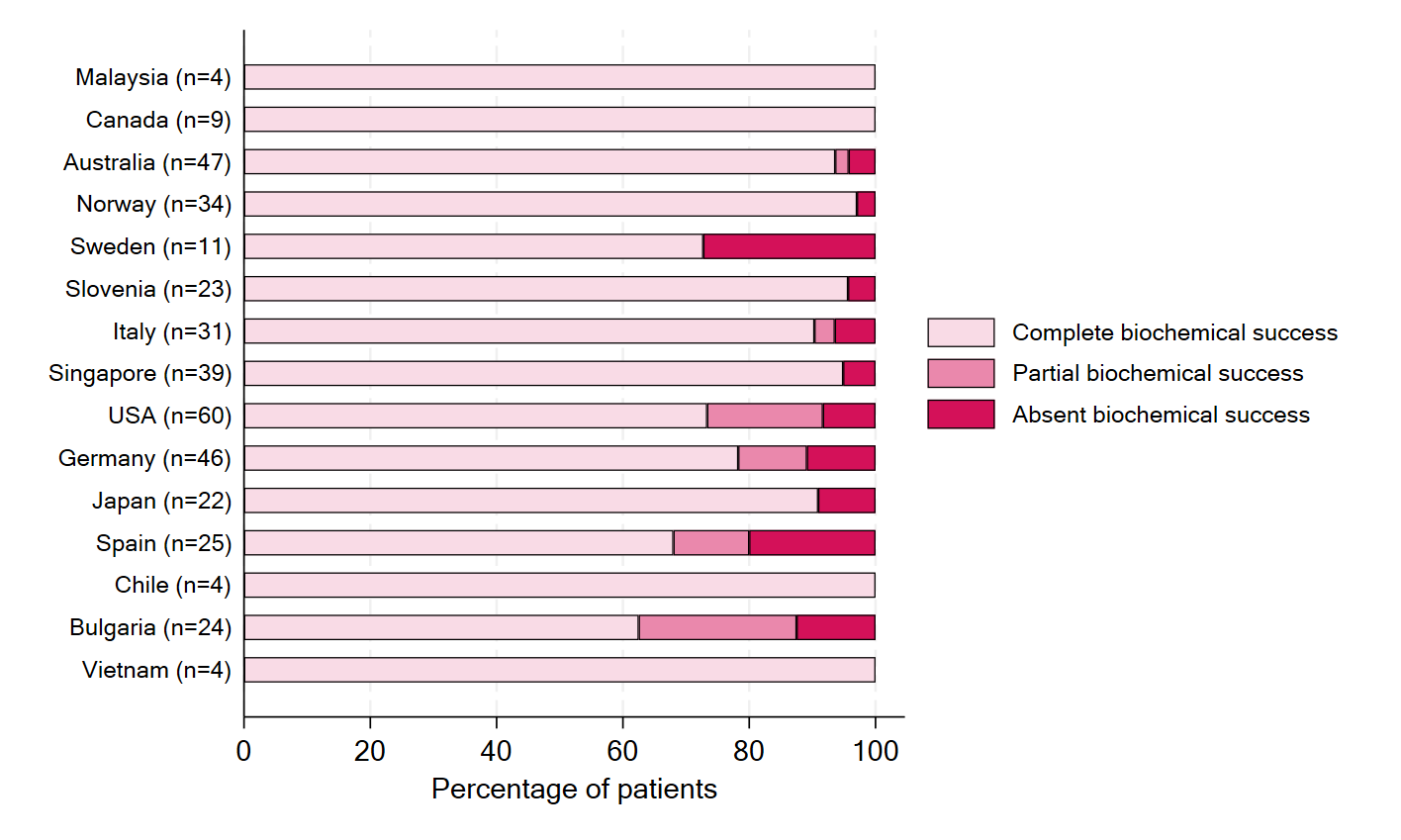


**Figure S4**. Horizontal stacked bar charts of percentage of patients with complete, partial, or absent success, by country of centre where adrenalectomy was performed, for patients with a minimum 180 days between adrenalectomy date and post-adrenalectomy blood pressure (or potassium, aldosterone, and renin) date. **A**. Clinical success. **B**. Biochemical success.

497/861 (57.7%) patients had available blood pressure, anti-hypertensive medication, and at least 180 days between adrenalectomy date and post-adrenalectomy blood pressure date to determine clinical success status. 383/861 (44.5%) patients had available potassium, aldosterone, renin, and at least 180 days between adrenalectomy date and post-adrenalectomy potassium, aldosterone, and renin dates to determine biochemical success status.

USA=United States of America.
